# Supplementary material for: IQ-Switch is a QF-based innocuous, silencing-free, and inducible gene switch system in zebrafish
Source: Commun Biol. 2021 Dec 16;4:1405. doi: 10.1038/s42003-021-02923-3 (PMC8677817; doi:10.1038/s42003-021-02923-3)
Supplement: Supplementary file 1 — Supplementary Information [file 42003_2021_2923_MOESM1_ESM.pdf]

Supplementary Figure 1

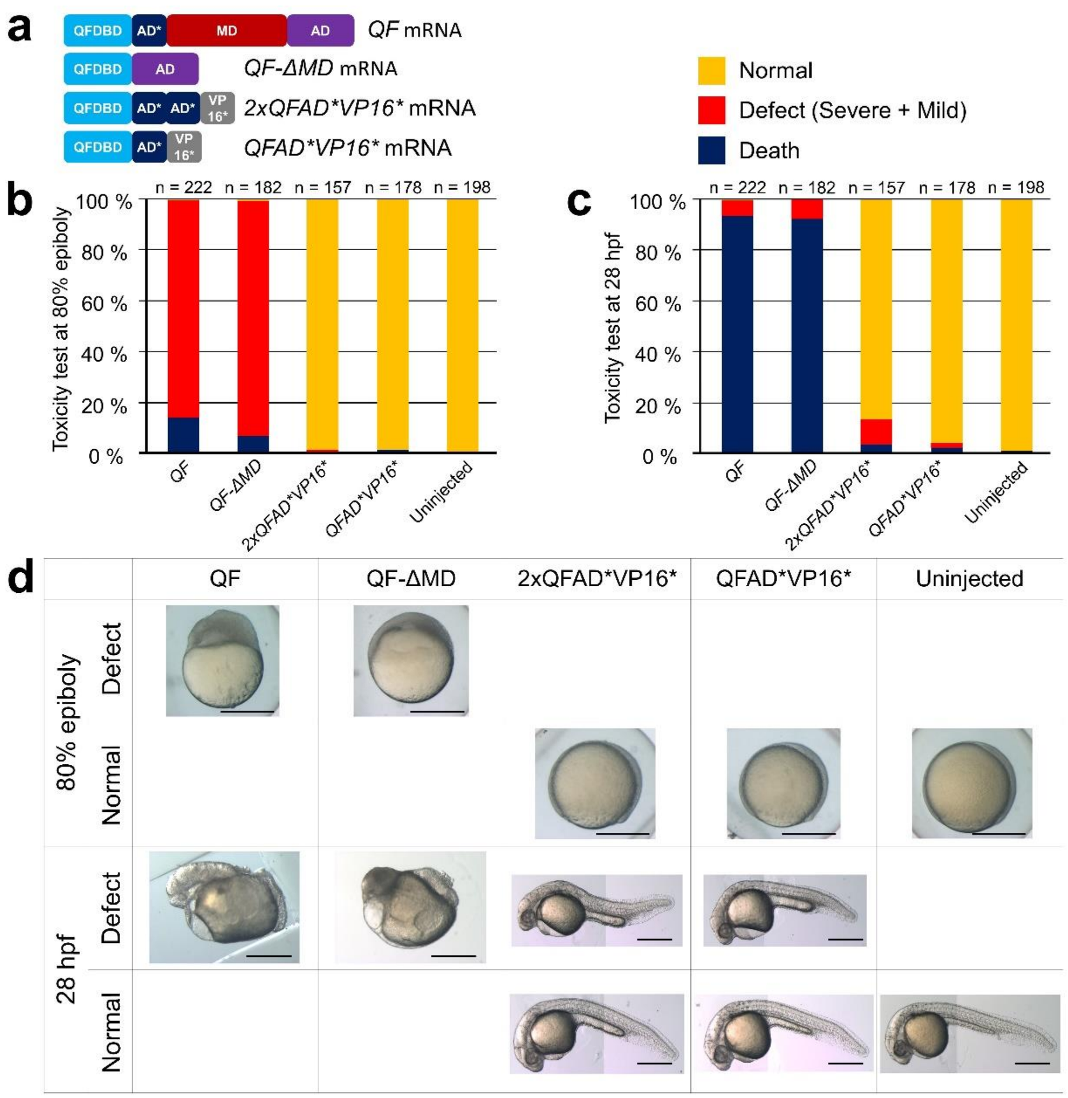

Supplementary Fig. 1 Embryonic toxicity of QF is originated in both the middle domain and carboxyl terminal activation domain. a Schematic diagram of driver constructs. After 50 pg of the *in vitro* transcribed mRNAs encoding indicated constructs were pressure injected into the 1 ~ 2 cell stage of embryos, the developmental defects were classified into three groups as normal, defect and death at 80% epiboly and 28 hpf. **b** The growth defects at 80% epiboly. **c** The growth defects at 28 hpf. **d** A representative image of each group at 80% epiboly and 28 hpf. Scale bar: 0.5 mm

Supplementary Figure 2

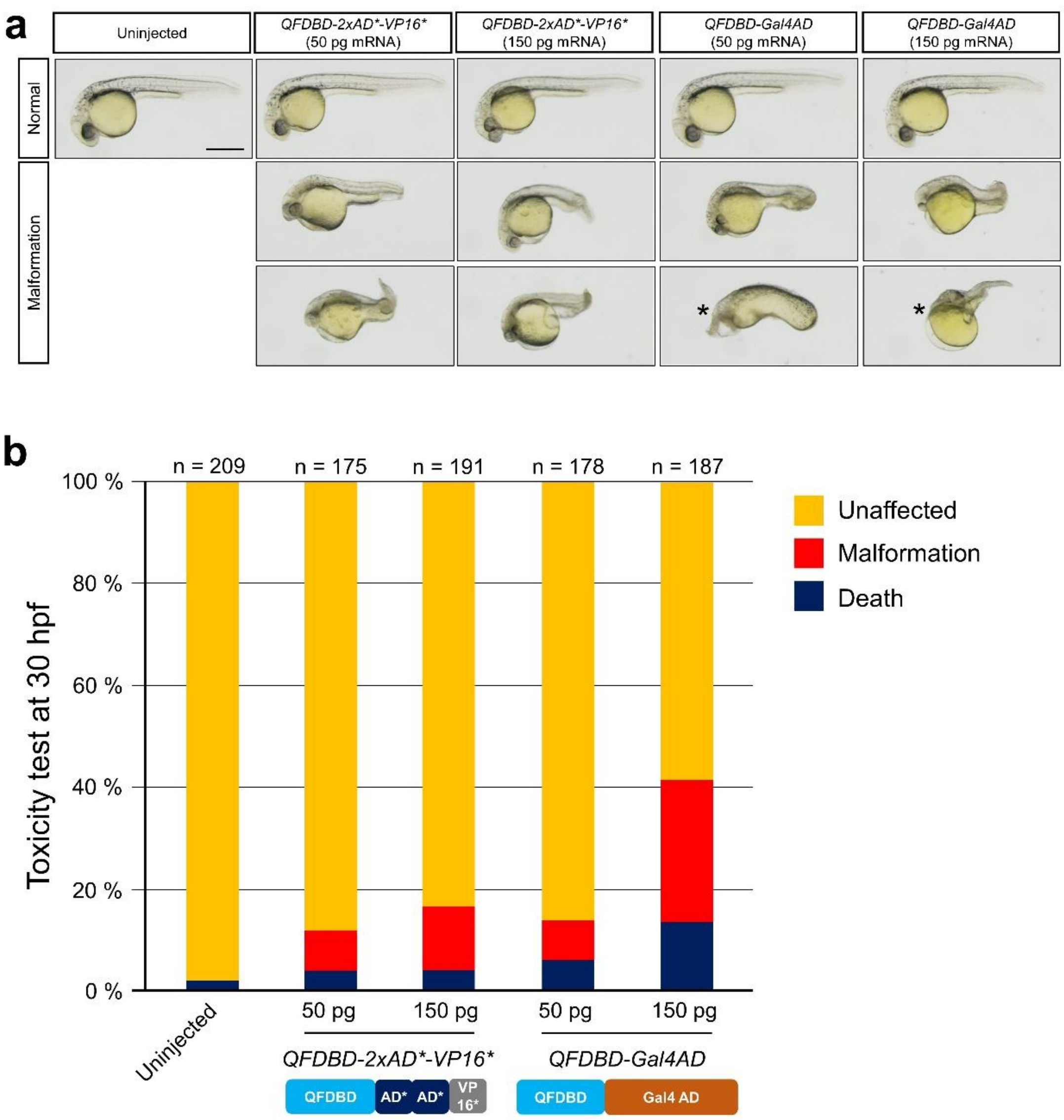

Supplementary Fig. 2 Comparison of driver toxicity. a The *in vitro* transcribed mRNA encoding *QFDBD-2xAD*-VP16** or *QFDBD-Gal4AD*[14] was pressure injected into 1–2 cell stage embryos using the indicated amount. Representative embryos are shown with various degrees of malformation at 30 hpf. Asterisks indicate the embryos with headless phenotype. **b** The embryonic morphology was classified as death, malformation, or normal at 30 hpf. Scale bar: 0.5 mm

Supplementary Figure 3

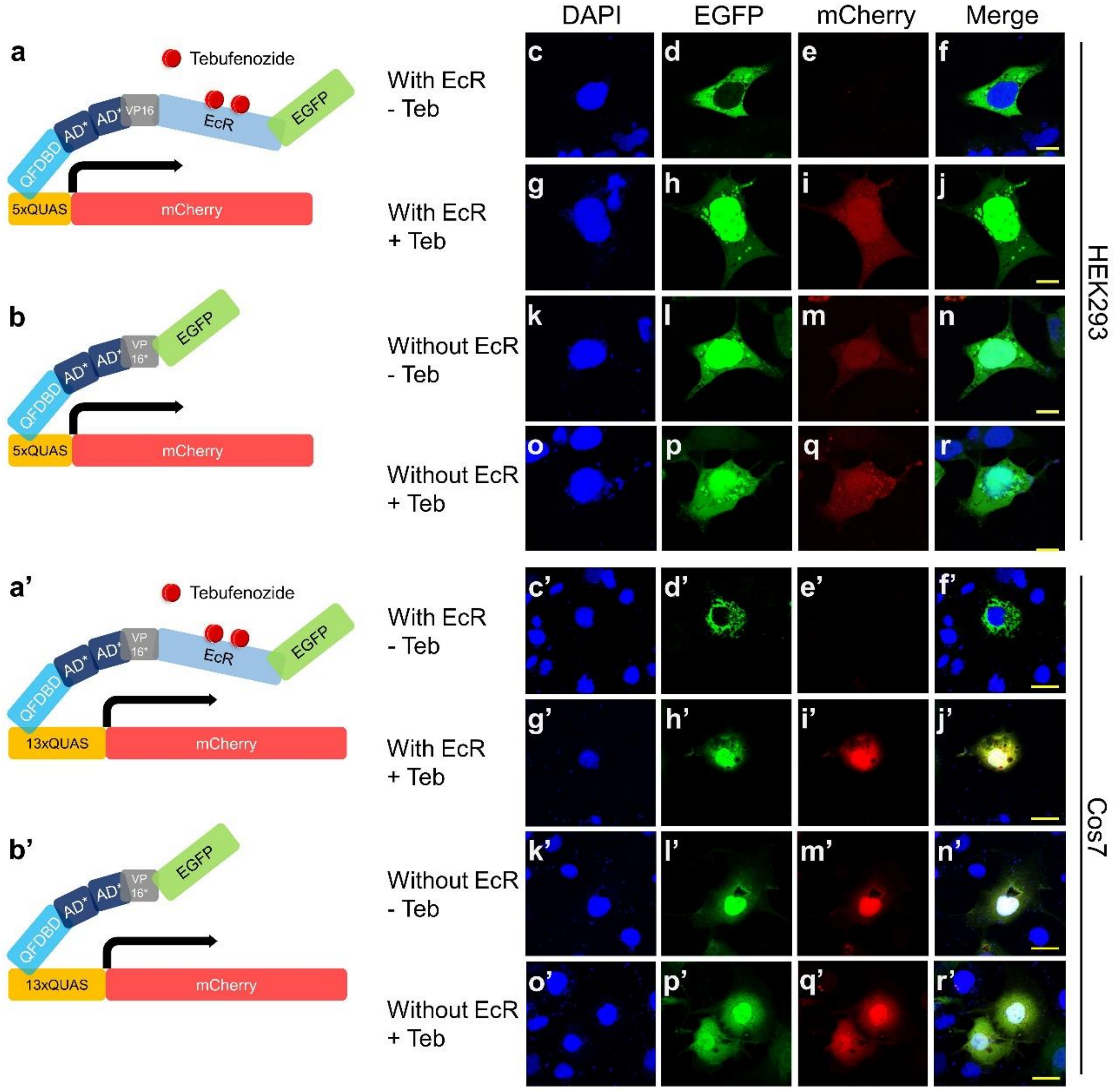

Supplementary Fig. 3 Subcellular localization of QFDBD-2xAD*-VP16*-EcR driver can be altered in mammalian cells by administration of Teb. **a–r** After HEK293 cells were transfected with the indicated plasmid constructs and then cultured for 24 h, 10 μM of Teb was added to the cell culture medium for 12 h before taking confocal images. **a** Schematic diagram of the mechanism of IQ-Switch for **c–j** The driver was tagged with EGFP, and the effector harbored *mCherry* reporter under the control of 5xQUAS. **c–f** In Teb(−) condition, the driver was exclusively localized in the cytosol, thus could not stimulate the effector. **g–j** With Teb, the driver translocated to the nucleus and stimulated *mCherry* expression. **b–r** An EcR-deleted driver was mainly localized in the nucleus where it constitutively activated the *mCherry* reporter regardless of Teb stimulation. **a'–r'** Identical experiments to **a–r** were carried out with

Cos7 cells using an effector plasmid encoding 13xQUAS instead of 5xQUAS. Note that without Teb stimulation, the driver of IQ-Switch equipped with a Teb-binding module and an EcR domain was exclusively tethered in the cytosol. The regulated cellular localization of the driver was absolutely dependent upon the existence of EcR and Teb stimulation. DAPI was used for labeling the nucleus. Abbreviations: Teb; tebufenozide. Scale bar: 100 μm

Supplementary Figure 4

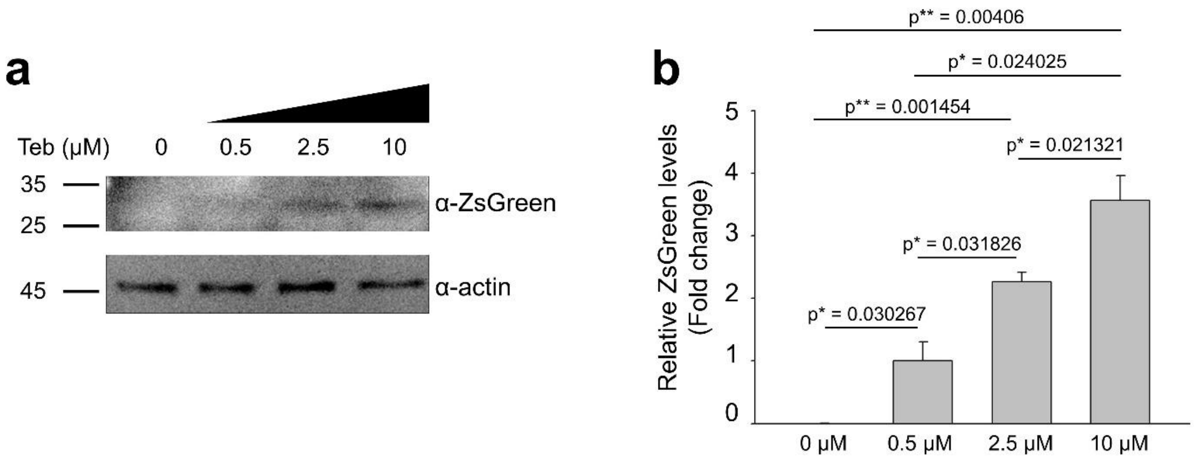

Supplementary Fig. 4 Teb dosage dependent responsiveness of IQ-Switch. a Larvae at 24 hpf collected from genetic crosses between F2 *Tg*(*ubb:QFDBD-2xAD*-VP16*-EcR*) driver and F2 *Tg*(*13xQUAS*:*ZsGreen-P2A*) effector strains were treated with indicated amount of Teb for 12 hours. Total embryonic lysates were separated on the 10% SDS-PAGE gel before subjected to western blotting with α-ZsGreen antibody. α-actin was used as a loading control.

b The relative expression level of ZsGreen was revised by in comparison to the amount of α-actin for the graphical visualization. Error bars stand for the standard deviation.

Supplementary Figure 5

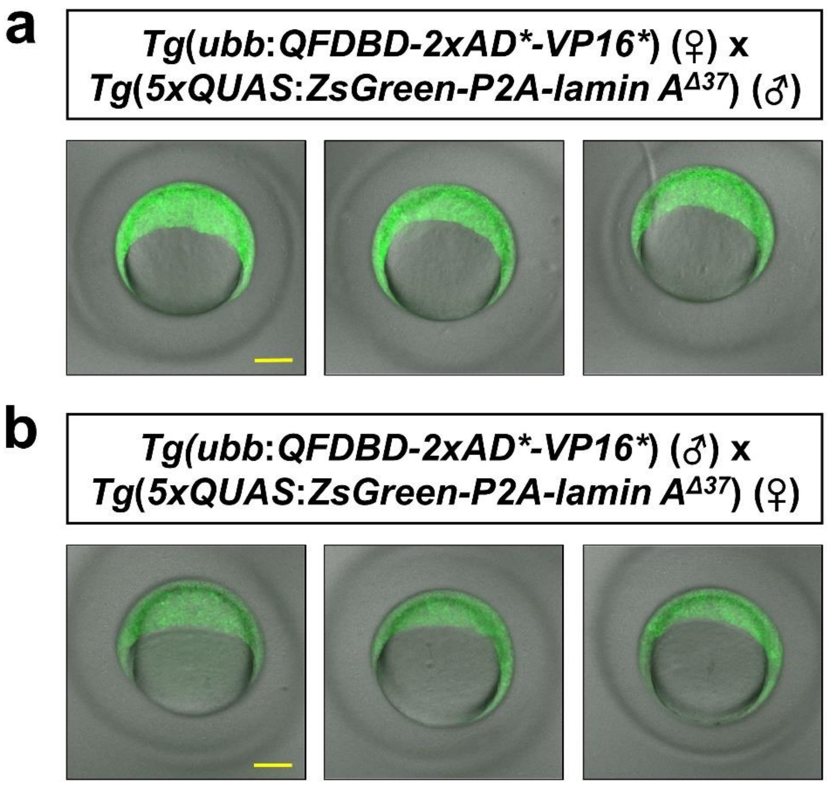

Supplementary Fig. 5 A constitutively active driver triggers transgene expression during early development without the aid of Teb stimulation. Three separate embryos were imaged under the confocal microscope at 6 hpf. Transgenic animals harboring a driver construct without EcR no longer require Teb stimulation for the induction of transgenes. **a** Genetic cross between F2 female *Tg*(*ubb:QFDBD-2xAD*-VP16**) and F2 male *Tg*(*5xQUAS:ZsGreen-P2A-lamin* $A^{\Delta37}$). **b** Genetic cross between F2 male *Tg*(*ubb:QFDBD-2xAD*-VP16**) and F2 female *Tg*(*5xQUAS:ZsGreen-P2A-lamin* $A^{\Delta37}$). Notably, embryos derived from female *Tg*(*ubb:QFDBD-2xAD*-VP16**) exhibited more intense ZsGreen fluorescence. This is likely due to the maternal deposition of QFDBD-2xAD*-VP16* transcriptional activator. Scale bar: 200 μm.

Supplementary Figure 6

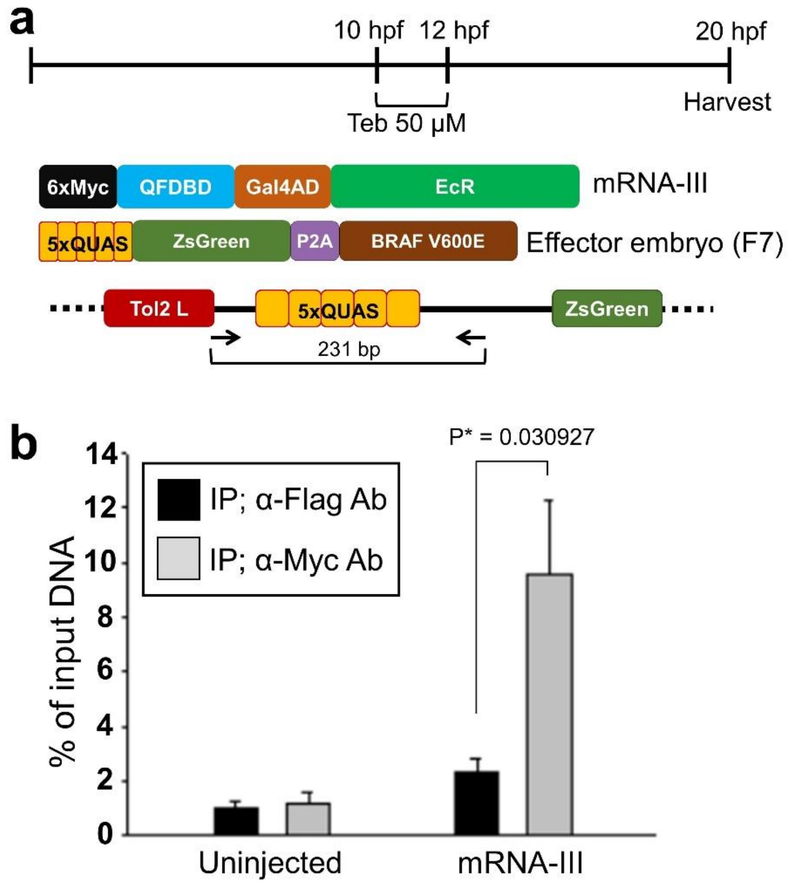

Supplementary Fig. 6 QFDBD may bind to QUAS in methylation independent manner. a Schematic diagram of ChIP experiments. The *in vitro* transcribed mRNA (50 pg) encoding 6x*Myc*-tagged *QFDBD-GAL4AD-EcR* was pressure injected into the 1 ~ 2 cell stage of embryos of F7 *Tg*(*5xQUAS*:*ZsGreen-P2A-BRAF*^{V600E}). After the injected embryos were exposed to 50 μM of Teb for 2 h at the early somite stage, they were harvested at 20 hpf. A primer set indicated with arrows amplifies 231 bp. **b** ChIP assay with anti-Myc and anti-Flag antibody. Uninjected embryos were used for the measurement of background ChIP enrichment. The anti-Flag antibody was also used as a negative control. The ChIP samples were subjected to qPCR with the primer set. q-PCR was accomplished in triplicate, and a p-value was shown in the panel. Error bars stand for the standard deviation.

Supplementary Figure 7

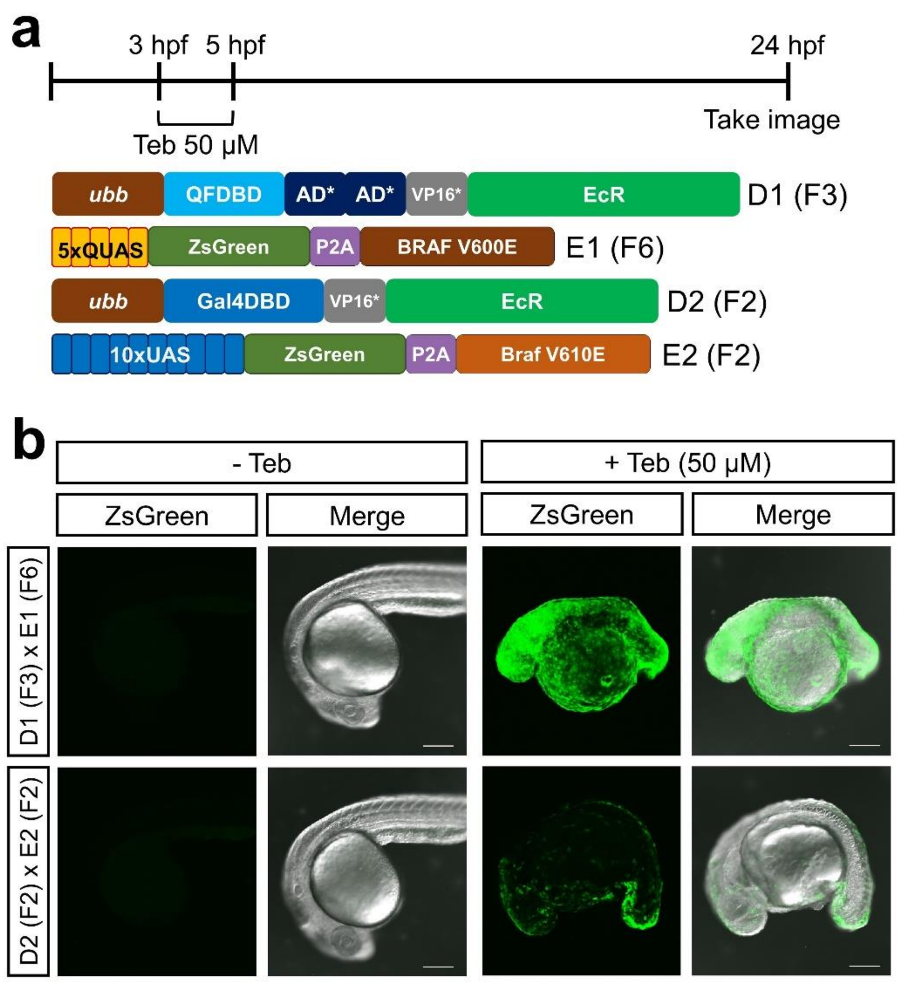

Supplementary Fig. 7 The resistance of IQ-Switch to transgene silencing over generations. a Schematic diagram of experimental procedure. The transgenic siblings were treated with 50 μM of Teb for 2 h before the onset of gastrulation and then observed under the confocal microscope at 24 hpf. **b** ZsGreen fluorescence of F7 siblings under the control of QF/5xQUAS was uniformly detected in the whole embryo (D1 + E1), whereas the same ZsGreen signal of as early as F3 embryos was observed only in limited tissues when driven by Gal4/10xUAS (D2 + E2). Abbreviations: Teb; tebufenozide. Scale bar; 200 μm.

Supplementary Figure 8

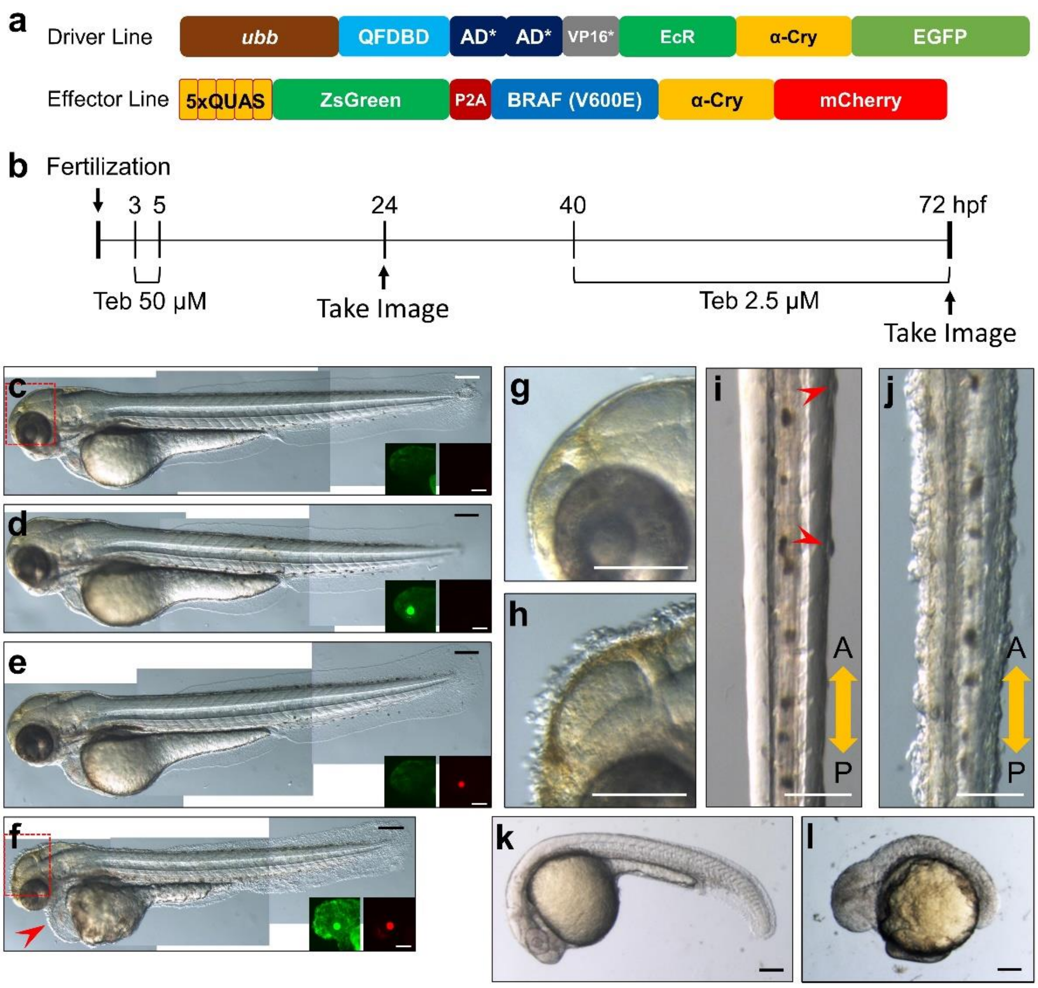

Supplementary Fig. 8 Ectopic expression of BRAF$^{(V600E)}$ during distinct developmental stages induces discrete embryonic malignances. a Driver and effector vector constructs for transgenesis. Zebrafish *α-crystallin* promoter was used to label the lens with green or mCherry fluorescent proteins as discernible markers. **b** Schematic diagram of the experimental design. **c–j** Live embryos treated with 2.5 µM of Teb from 40 hpf to 72 hpf after a genetic cross of heterozygous driver and effector lines. **c–f** Inserts are image clippings showing that fluorescence is focused in the lens. **c** WT embryo, which expressed neither EGFP nor mCherry in the lens (N = 24). **d** *Tg*(*ubb*:*QFDBD-2xAD*-VP16*-EcR*) with only EGFP in the lens (N = 24). **e** *Tg*(*5xQUAS*:*ZsGreen-P2A-BRAF*^{V600E}) with only mCherry-positive lens (N = 21). **f** An embryo harboring both *ubb*:*QFDBD-2xAD*-VP16*-EcR* and *5xQUAS*:*ZsGreen-P2A-BRAF*^{V600E} with simultaneous expression of EGFP and mCherry in the lens (N = 22). Red arrowhead indicates cardiac defects with inflated pericardial sac. **g** Magnified brain image of red dotted line in (**c**). The facial contour of the embryo was smooth and sleek. **h** Magnified

brain image of red broken line in (**f**). Indented epithelium of the brain was a characteristic feature of embryos exhibiting elevated BRAF$^{(V600E)}$ expression. **i** Magnified overview image of trunk in (**c**). Red arrowheads indicate discernable neuromasts. **j** Enlarged overview image of trunk in (**f**). Fluffy structure of the trunk made neuromasts indistinguishable. **k, l** After exposure of embryos to a high dosage of Teb (50 μM) in a brief period of time (2 h) before the onset of gastrulation, the embryos were raised in Teb-free embryo culture medium until they reached 24 hpf. **k** WT embryos grew normally irrespective of Teb treatment. (**l**) A transgenic embryo that elicited ZsGreen reporter expression at 24 hpf (fig. 2e) developed abnormally without tail and eyes with 100% penetrance (N = 28). Abbreviations: Teb; tebufenozide, A; anterior, P; posterior. Scale bar: 100 μm.

Supplementary Figure 9

5x QUAS

GGGTAATCGCTTATCCTCGGATAAACAATTATCCTCACGGGTAATCGCTTATCCGCTCGGGTAA
TCGCTTATCCTCGGGTAATCGCTTATCCTTGCAAGGGTCGACTCTAGAGGGTATATAATGGATCCC
ATCGCGTCTCAGCCTCACTTTGAGCTCCTCCACACGAATTCCCTCGACCTCGAAGACGCGT

9x QUAS

GGGTAATCGCTTATCCTCGGATAAACAATTATCCTCACGGGTAATCGCTTATCCGCTCGGGTAA
TCGCTTATCCTCTCGGGTAATCGCTTATCCTCGGATAAACAATTATCCTCACGGGTAATCGCTTA
TCCGCTCGGGTAATCGCTTATCCTCGGGTAATCGCTTATCCTTGCAAGGGTCGACTCTAGAGGGT
ATATAATGGATCCCATCGCGTCTCAGCCTCACTTTGAGCTCCTCCACACGAATTCCCTCGACCTCGAA
GACGCGT

13x QUAS

GGGTAATCGCTTATCCTCGGATAAACAATTATCCTCACGGGTAATCGCTTATCCGCTCGGGTAA
TCGCTTATCCTCGGGTAATCGCTTATCCTCGGGTAATCGCTTATCCTCGGATAAACAATTATCCT
CACGGGTAATCGCTTATCCGCTCGGGTAATCGCTTATCCTCGGATAAACAATTATCCTCACGGG
TAATCGCTTATCCGCTCGGGTAATCGCTTATCCTCGGGTAATCGCTTATCCTTGCAAGGGTCGAC
TCTAGAGGGTATATAATGGATCCCATCGCGTCTCAGCCTCACTTTGAGCTCCTCCACACGAATTCCC
TCGACCTCGAAGACGCGT

17x QUAS

GGGTAATCGCTTATCCTCGGATAAACAATTATCCTCACGGGTAATCGCTTATCCGCTCGGGTAA
TCGCTTATCCTCGGGTAATCGCTTATCCTCGGGTAATCGCTTATCCTCGGGTAATCGCTTATCCT
CGGATAAACAATTATCCTCACGGGTAATCGCTTATCCGCTCGGGTAATCGCTTATCCTCGGATA
AACAATTATCCTCACGGGTAATCGCTTATCCGCTCGGGTAATCGCTTATCCTCGGATAAACAAT
TATCCTCACGGGTAATCGCTTATCCGCTCGGGTAATCGCTTATCCTCGGGTAATCGCTTATCCTT
GCAAGGGTCGACTCTAGAGGGTATATAATGGATCCCATCGCGTCTCAGCCTCACTTTGAGCTCCTCC
ACACGAATTCCCTCGACCTCGAAGACGCGT

qa-2 (bold) qa-1F-qa-1S Zebrafish *β-actin* minimal promoter

Supplementary Fig. 9 Sequence information of the element regions of effector cassettes encoding different numbers of QUAS sequences. 5xQUAS represented an original configuration of five tandem repeats of 16-bp length of QUAS where a single copy of the qa-1F-qa-1S variant (bold red) is located immediately behind the first qa-2 (bold black). The other three qa-2-binding sites follow the qa-1F-qa-1S[14,15]. The 9x, 13x, and 17x QUAS contain different numbers of qa-1F-qa-1S variant, from 2, 3, and 4, respectively. Zebrafish *β-actin* minimal promoter is labeled with bold green type.

Supplementary Figure 10

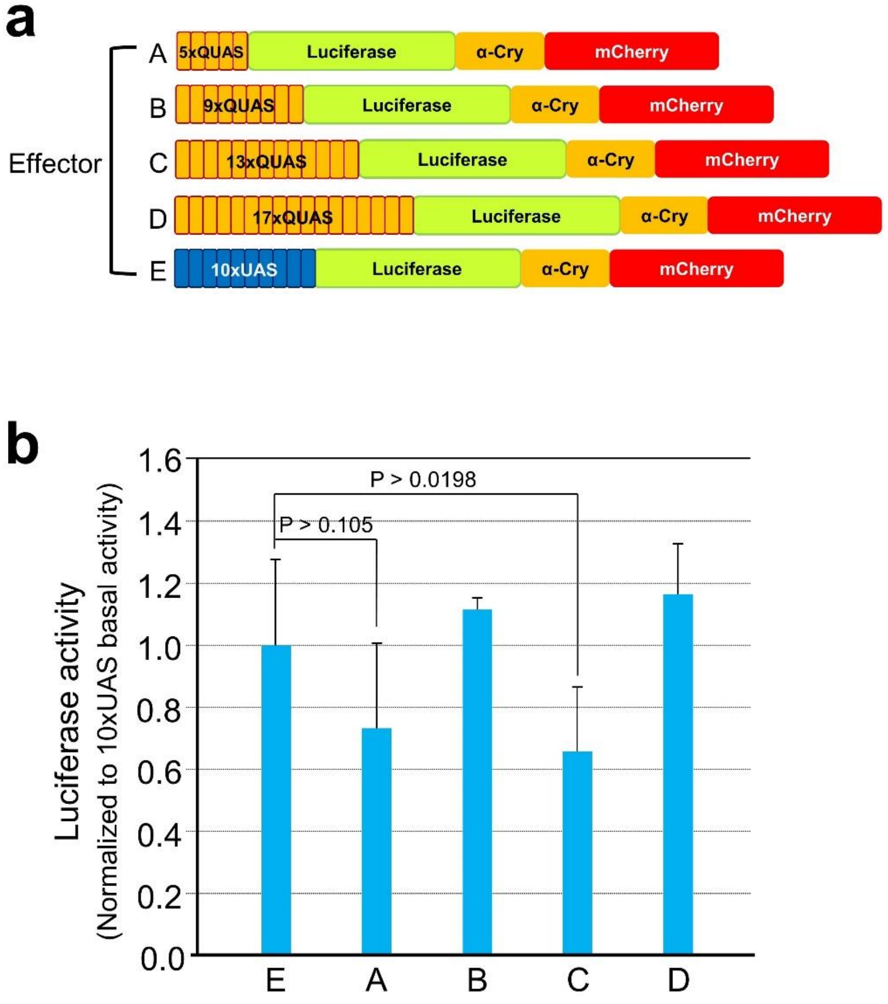

Supplementary Fig. 10 IQ-Switch is not leaky. a Schematic diagram of effector constructs. **b** Basal leakiness of each individual QUAS effector was compared with 10xUAS; no significant differences were observed. Error bars stand for the standard deviation.

Supplementary Figure 11

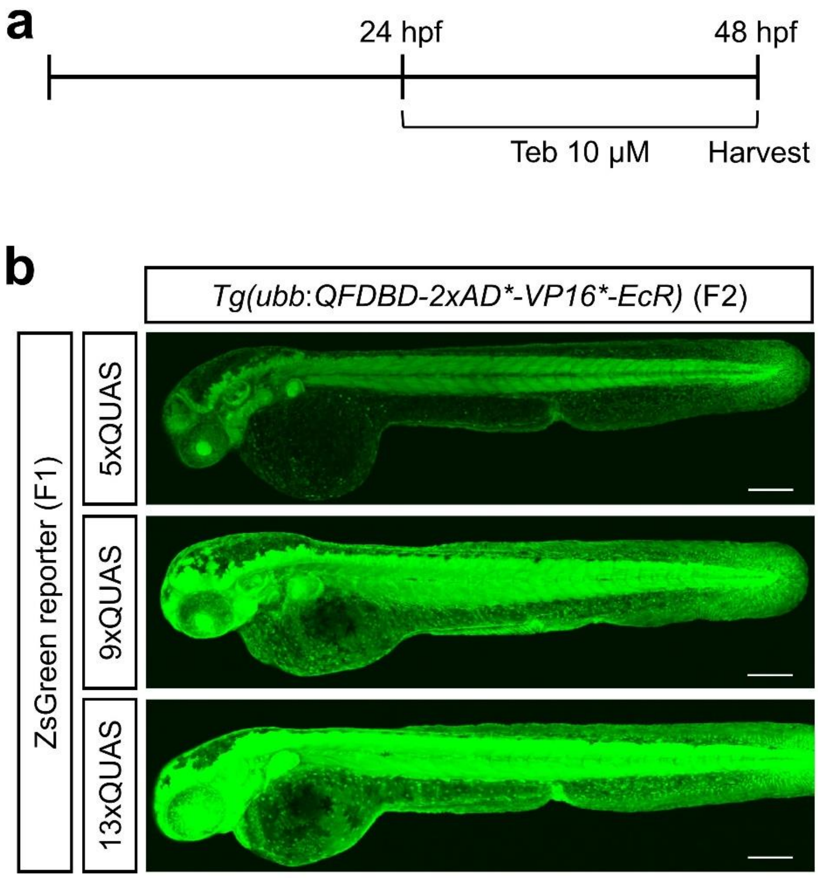

Supplementary Fig. 11 Teb dosage-dependent induction levels of a transgene. a Schematic diagram of the experimental procedure. **b** An F2 driver of *Tg*(*ubb*:*QFDBD-2xAD*-VP16*-EcR*) was subjected to mating with discrete F1 Tg lines equipped with ZsGreen reporter having different numbers of QUAS repeats as indicated. After exposing the embryos to a low dose of Teb (10 μM) for 24 h, ZsGreen fluorescence was observed under the confocal microscope. Note that in contrast to supplementary figure 13 where embryos treated with 50 μM of Teb showed much stronger expression of ZsGreen, being completely saturated at over 9xQUAS, the ZsGreen only reached saturation in embryos with 13xQUAS when they were exposed to 10 μM of Teb. Abbreviations: Teb; tebufenozide. Scale bar: 200 μm.

Supplementary Figure 12

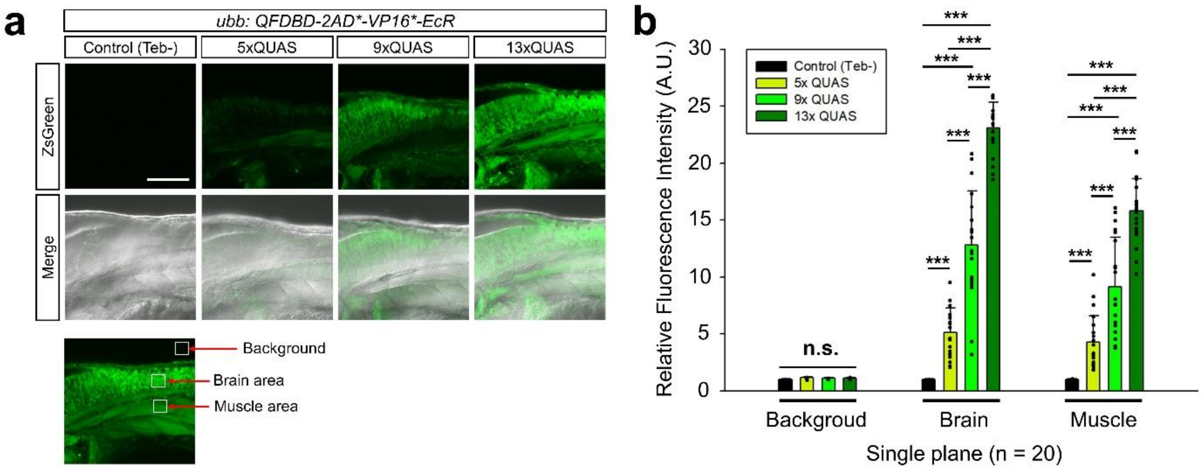

Supplementary Fig. 12 Quantification of a single plane fluorescence image. a Representative single plane confocal image of larvae at 3 dpf exposed to 10 μM of Teb for 24 h. **b** Fluorescent signals in the brain and muscle were evaluated in comparison with that of background (-Teb) whose intensity was assumed as 1 arbitrary unit. A single plane confocal image with a fixed confocal setting from randomly collected embryos (n = 20) was used to measure the fluorescent signal of ZsGreen under the control of 5x, 9x, and 13xQUAS. P value indicated with (***) stands for < 0.001. Error bars stand for the standard deviation. Abbreviations: A.U.; arbitrary unit, n.s.; not significant. Scale bar: 100 μm.

Supplementary Figure 13

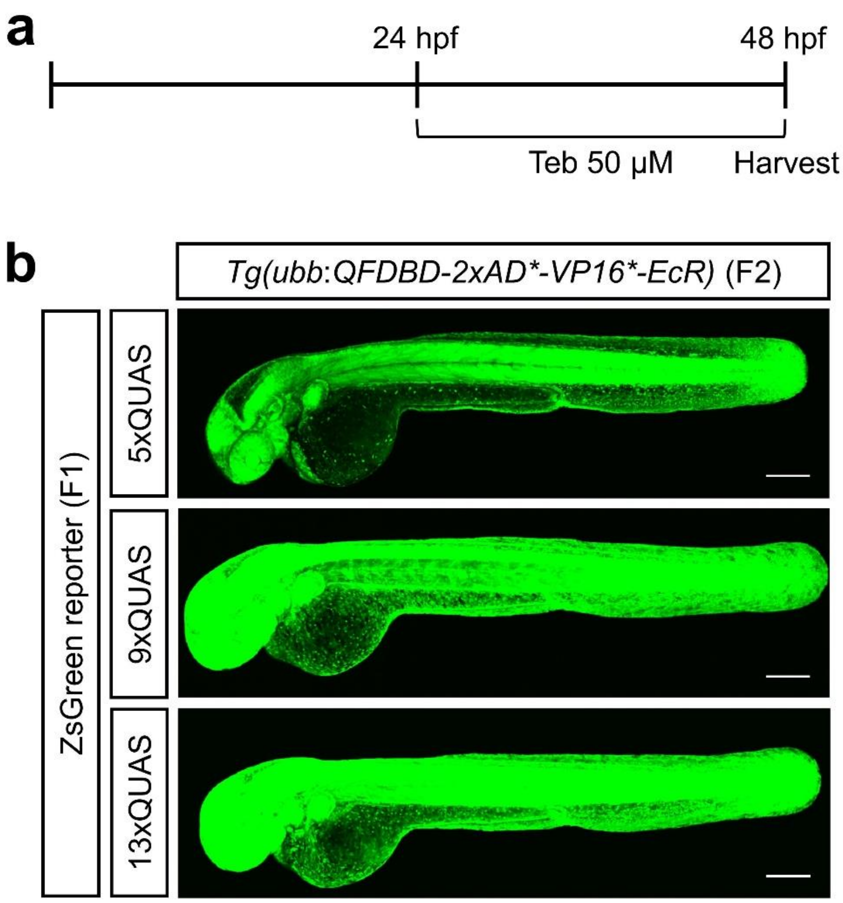

Supplementary Fig. 13 High dose of Teb treatment leveled off ZsGreen fluorescent signal from beyond the 9xQUAS. a Schematic drawing of the experimental procedure. **b** Genetic cross of the F2 driver with the indicated F1 effector lines. The transgenic siblings were exposed to 50 μM of Teb at 24 hpf, then fixed at 48 hpf for confocal imaging. The strength of ZsGreen reporter was gradually elevated by increasing the number of QUAS repeats though, at 50 μM of Teb, ZsGreen intensity became saturated from beyond 9xQUAS under the confocal microscope with a fixed setting. Scale bar: 200 μm

Supplementary Figure 14

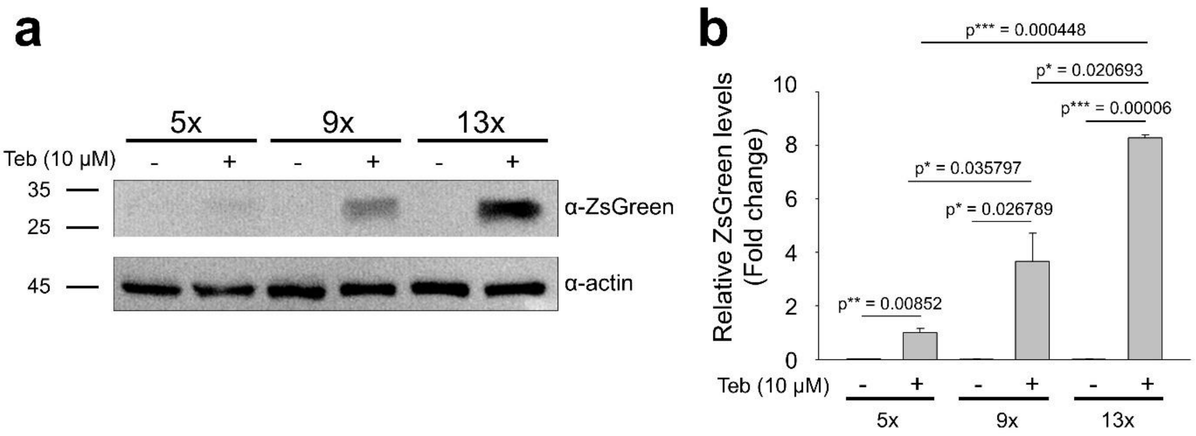

Supplementary Fig. 14 Evaluation of Teb responsiveness of embryos with different copies of QUAS elements. Larvae obtained from genetic crosses between a F3 *Tg*(*ubb:QFDBD-2xAD*-VP16*-EcR*) with individual F2 *Tg*(*5xQUAS:ZsGreen-P2A*), *Tg*(*9xQUAS:ZsGreen-P2A*) and *Tg*(*13xQUAS:ZsGreen-P2A*) were randomly collected for the subsequent assay. **a** Embryos at 1 dpf were administered with 10 μM of Teb for 24 hours, and then subjected to western blotting using anti-ZsGreen antibody. α-actin was used as a loading control. **b** The level of ZsGreen was quantified after adjustment in comparison with that of α-actin. Error bars stand for the standard deviation.

Supplementary Figure 15

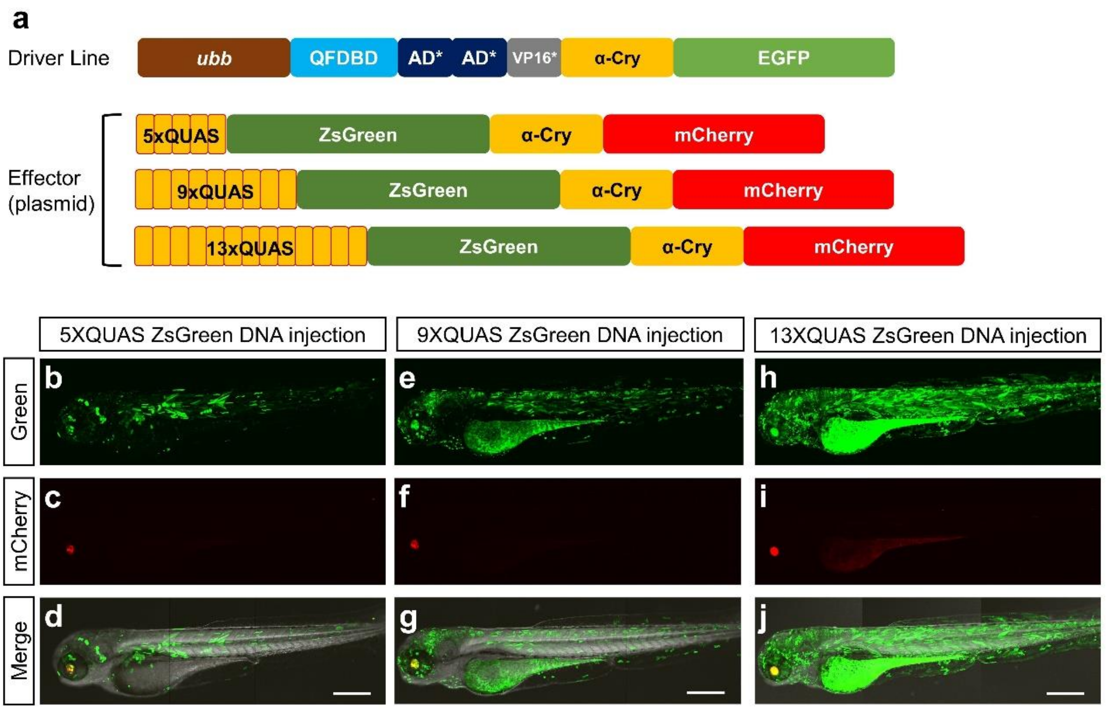

Supplementary Fig. 15 Extension of QUAS tandem repeats is positively correlated with fluorescence intensity of the ZsGreen reporter. a Schematic diagram of driver zebrafish and corresponding effector plasmid configurations. **b–j** *Tg*(*ubb*:*QFDBD-2xAD*-VP16**), an F2 driver line without EcR, was crossed with a WT sibling; their offspring were subjected to forced injection of 10 pg of effector plasmids. When the injected larvae reached 72 hpf, EGFP fluorescence intensity was analyzed under the confocal microscope. mCherry labeled the lens with red color as a molecular indicator for the actual delivery of effector plasmids into the embryo. Note that increasing copies of QUAS from 5 to 13 intensified the EGFP fluorescence.

Scale bar: 200 μm.

Supplementary Figure 16

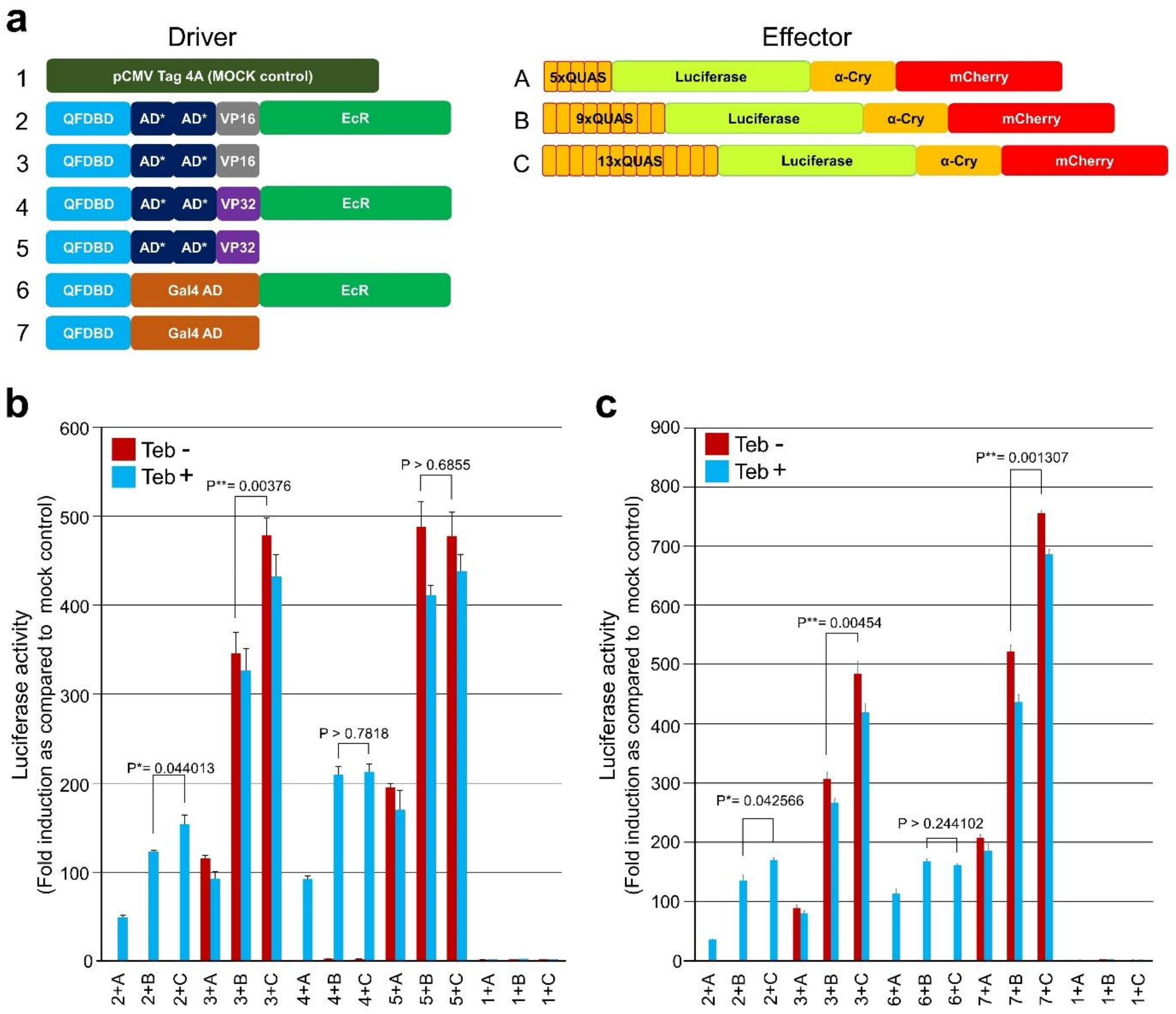

Supplementary Fig. 16 Comparison of the transgene-inducing capabilities in QF-2xAD*-VP16*, QF-2xAD*-VP32*, and QF-Gal4 (QF-Gal4AD). a Configuration of driver and effector constructs for the measurement of luciferase activity. Individual effectors including mock control were labeled with Arabic numbers while effectors following the order of QUAS repeats were labeled alphabetically. The combined driver and effector were co-transfected into HEK293 cells, and then cultured for 4 h before treatment with 10 μM of Teb. After 24 h of further cultivation, the transfectants were harvested for dual luciferase assay. **b** Comparison of QFDBD-2xAD*-VP16* variants with constructs of QFDBD-2xAD*-VP32. Note that both inducible (4) and constitutively active (5) forms of drivers with QFDBD-2xAD*-VP32 reached a plateau at more than 9xQUAS. **c** Comparison of variants of QFDBD-2xAD*-VP16* and QFDBD-Gal4AD. Note that the constitutively active QFDBD-Gal4AD driver (7) magnified the luciferase activity in line with the increased numbers of QUAS. However, the QFDBD-Gal4AD-

EcR (6) by Teb stimulation reached the maximum as early as 9xQUAS, whose activity was comparable to the strength driven by QFDBD-2xAD*-VP16*-EcR with 13xQUAS. Luciferase activity was measured in triplicate experiments, and standard deviation is shown in each panel.

Supplementary Figure 17

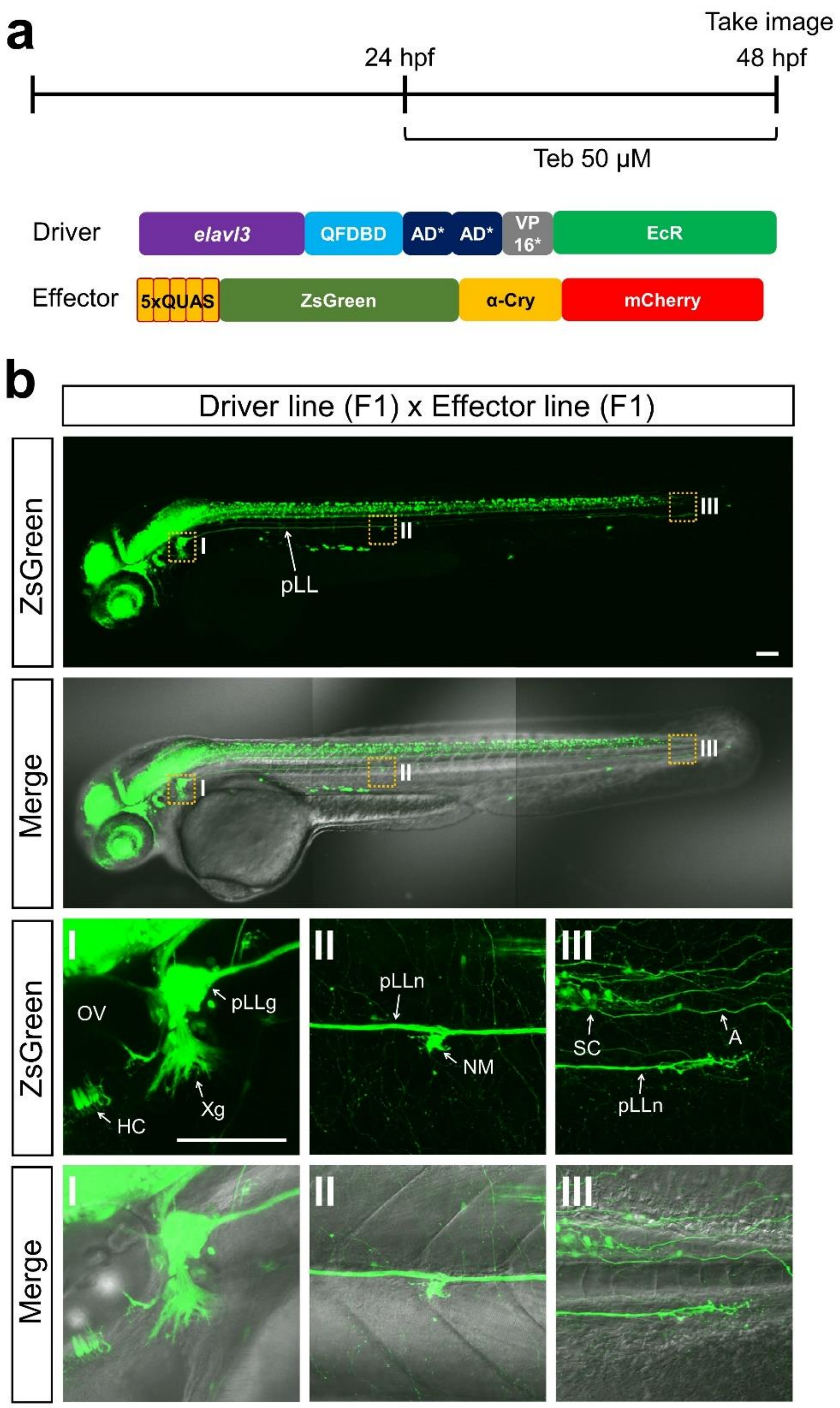

Supplementary Fig. 17 Tissue-specific transgene induction can be achieved by Teb stimulation. a Schematic diagram of the experimental scheme. Heterozygous F1 driver and F2 effector lines equipped with the indicated constructs were crossed to obtain embryos that were exposed to Teb (50 μM) for one day after 24 hpf. **b** Lateral view of a representative

embryo at 48 hpf. ZsGreen reporter was exclusively detected in pan-neuronal tissues including the posterior lateral line system (pLL, dotted boxes) where the ZsGreen positive domains are completely overlapped with the previously reported expression pattern of endogenous *elavl3*[38,39]. **I–III** Magnified images of dotted lines in (**b**) stand for the outgrowth of pLL nerve from pLL ganglion innervating the neuromasts. Abbreviations: A; axonal projection, HC; ear hair cells, NM; neuromasts, OV; otic vesicle; pLL; posterior lateral line; pLLg; pLL ganglion, pLLn; pLL nerve; SC; spinal cord, Teb; tebufenozide, Xg; vagal ganglion. Scale bar: 100 μm.

Supplementary Figure 18

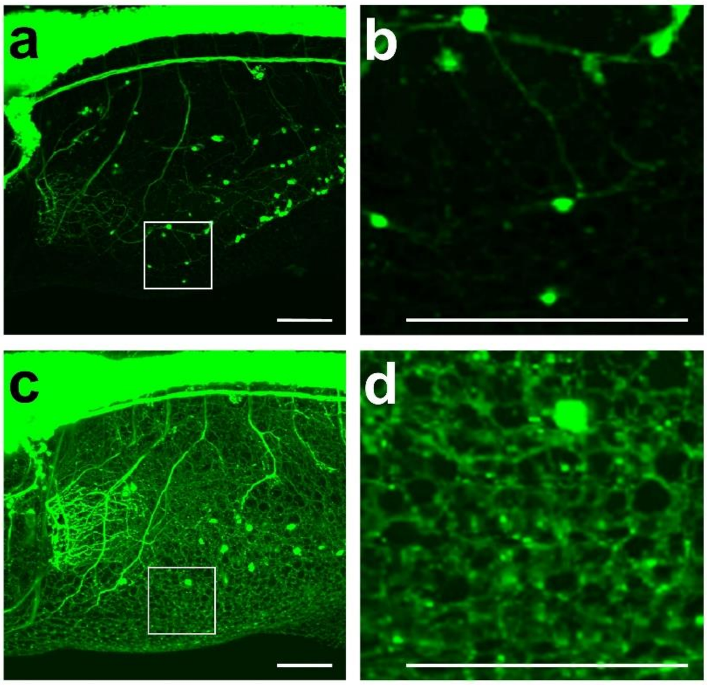

Supplementary Fig. 18 ZsGreen became gradually obvious in ELY sensory neurons.

Representative image of embryos in fig. 6a at 5 dpf. **a** Confocal image of an embryo derived from F2 *Tg(elavl3:QFDBD-2AD*-VP16*)* mated with F3 *Tg*(*5xQUAS*:*ZsGreen-P2A*). **b** Magnified image of white open square in (**a**). **c** Confocal image of a larva obtained from a genetic cross between *Tg(elavl3:QFDBD-2AD*-VP16*)* and *Tg*(*13xQUAS*:*ZsGreen-P2A*). **d** Enlarged image of white open square in (**c**). Note that the web-like fluorescent signal in the ELY is most apparent in the double transgenic embryo with only the 13xQUAS element. Scale bar: 200 μm.

Supplementary Table 1

	Inducible driver configuration	Toxicity	Leakiness	Sensitivity to Teb	References
1	QFDBD – AD* – MD – AD – EcR	+++++	+/-	++++	[13],[19],[21],[26]
2	QFDBD – AD – EcR	++++	++	+++++	[13],[19],[21],[26]
3	QFDBD – AD* – AD* – VP16* – EcR	-	-	+++	
4	QFDBD – AD* – AD* – EcR	NT	-	+	
5	QFDBD – AD* – VP16* – VP16* – EcR	NT	-	++	
6	QFDBD – AD* – VP16 – EcR	NT	+++	++++	
7	QFDBD – AD* – VP16* – EcR	NT	-	++	
8	QFDBD – AD* – EcR	NT	-	+/-	
9	QFDBD – VP16* – EcR	NT	-	-	
10	QFDBD – AD* – AD* – VP16* – VP16* – EcR	+	+++++	+/-	
11	QFDBD – AD* – AD* – AD* – VP16* – EcR	+	-	+++	
12	QFDBD – AD* – AD* – VP64 – EcR	+++	+++++#	+/-#	
13	QFDBD – AD* – AD* – VP32 – EcR	+*	+/-	+++	
14	QFDBD – Gal4 AD – EcR	+/-**	-	++++	[14]
15	GAL4DBD – VP16* – EcR	-	-	+++	[2],[5]

Supplementary Table 1. Driver vector configurations. Toxicity was tested by injecting 50 pg of *in vitro* transcribed mRNA into wild-type (WT) embryos. Driver toxicity was determined by observing the frequency of embryonic malformation after injecting 50–150 pg of *in vitro* transcribed mRNA at the 1–2 cell stage. An asterisk (*) indicates the inherited toxicity of a driver labeled with the Arabic number 13 where the embryos look normal when they received 50 pg of *in vitro* transcribed mRNA, however none of the transgenic lines (five lines tested) generated with the driver could be maintained beyond F0 due to the emergence of edema, which progressively accumulated after 2 dpf of F1 embryos. Double asterisks (**) indicate potential toxicity of QFDBD-Gal4AD (Arabic number 14), which was not evident when injected with 50 pg of its mRNA, as is the case with QFDBD-2xAD*-VP16* (Arabic number 3). However, by increasing the dosage of mRNA to 150 pg, a large proportion of *QFDBD-Gal4AD* mRNA-delivered embryos showed distinctive embryonic malformations whose phenotypes were never observed in the *QFDBD-2xAD*-VP16** mRNA-injected group (Supplementary Fig. 1). Transgene leakiness and Teb responsiveness were quantified by measuring luciferase

reporter activity, in addition to a sample marked with #, which was analyzed by observing ZsGreen reporter expression under a fluorescence microscope after injecting 10 pg of plasmid DNA, and labeled with the Arabic numeral 12. NT; not tested. The strength of the discrete criterion was depicted with a plus/minus sign. (−) none detectable, (+/−) negligible but detectable, (+) detectable, (++) moderate, (+++) high, (++++) higher, (+++++) highest.

Supplementary Table 2

Driver	pQFDBD-AD*-MD-AD, α-cry:EGFP
	pQFDBD-AD*-MD-AD-EcR, α-cry:EGFP
	pQFDBD-AD, α-cry:EGFP
	pQFDBD-AD-EcR, α-cry:EGFP
	pQFDBD-2x AD*-VP16*, α-cry:EGFP
	pQFDBD-2x AD*-VP16*-EcR, α-cry:EGFP
	pQFDBD-2x AD*-EcR, α-cry:EGFP
	pQFDBD-AD*-2x VP16*-EcR, α-cry:EGFP
	pQFDBD-AD*-VP16-EcR, α-cry:EGFP
	pQFDBD-AD*-VP16*-EcR, α-cry:EGFP
	pQFDBD-AD*-EcR, α-cry:EGFP
	pQFDBD-VP16*-EcR, α-cry:EGFP
	pQFDBD-2x AD*-2x VP16*-EcR, α-cry:EGFP
	pQFDBD-3x AD*-VP16*-EcR, α-cry:EGFP
	pQFDBD-2x AD*-VP32-EcR, α-cry:EGFP
	pQFDBD-2x AD*-VP64-EcR, α-cry:EGFP
	pQFDBD-Gal4AD, α-cry:EGFP
	pQFDBD-Gal4AD-EcR, α-cry:EGFP
	pubb-QFDBD-AD*-MD-AD, α-cry:EGFP
	pubb-QFDBD-AD*-MD-AD-EcR, α-cry:EGFP
	pubb-QFDBD-AD, α-cry:EGFP
	pubb-QFDBD-AD-EcR, α-cry:EGFP
	pubb-QFDBD-2x AD*-VP16*, α-cry:EGFP
	pubb-QFDBD-2x AD*-VP16*-EcR, α-cry:EGFP
	pubb-QFDBD-2x AD*-EcR, α-cry:EGFP
	pubb-QFDBD-AD*-2x VP16*-EcR, α-cry:EGFP
	pubb-QFDBD-AD*-VP16-EcR, α-cry:EGFP
	pubb-QFDBD-AD*-VP16*-EcR, α-cry:EGFP
	pubb-QFDBD-AD*-EcR, α-cry:EGFP
	pubb-QFDBD-VP16*-EcR, α-cry:EGFP
	pubb-QFDBD-2x AD*-2x VP16*-EcR, α-cry:EGFP
	pubb-QFDBD-3x AD*-VP16*-EcR, α-cry:EGFP
	pubb-QFDBD-2x AD*-VP32-EcR, α-cry:EGFP
	pubb-QFDBD-2x AD*-VP64-EcR, α-cry:EGFP
	pubb-QFDBD-Gal4AD, α-cry:EGFP
	pubb-QFDBD-Gal4AD-EcR, α-cry:EGFP
	pelav3-QFDBD-2x AD*-VP16*, α-cry:EGFP
	pelav3-QFDBD-2x AD*-VP16*-EcR, α-cry:EGFP
Effector	p5xQUAS-ZsGreen-P2A, α-cry:EGFP
	p5xQUAS-ZsGreen-P2A, α-cry:mCherry
	p9xQUAS-ZsGreen-P2A, α-cry:mCherry
	p13xQUAS-ZsGreen-P2A, α-cry:mCherry
	p10xUAS-ZsGreen-P2A, α-cry:EGFP
	p10xUAS-ZsGreen-P2A, α-cry:mCherry
	p10xUAS-ZsGreen-P2A-Braf V610E, α-cry:EGFP
	p5xQUAS-ZsGreen-P2A-BRAF V600E, α-cry:EGFP

	p5xQUAS-ZsGreen-P2A-BRAF V600E, α-cry:mCherry
	p5xQUAS-ZsGreen-P2A-lamin $A^{\Delta 37}$, α-cry:EGFP
	p5xQUAS-mCherry, α-cry:mCherry
	p9xQUAS-mCherry, α-cry:mCherry
	p13xQUAS-mCherry, α-cry:mCherry
	p5xQUAS-MCS, α-cry:mCherry
	p9xQUAS-MCS, α-cry:mCherry
	p13xQUAS-MCS, α-cry:mCherry
**Backbone plasmid**	pKY64-miniTol2-opt-CRY-eGFP

Supplementary Table 2. Plasmid constructs for binary IQ-Switch transgenesis. All the effector and driver plasmids were generated based on a single backbone plasmid, designated pKY64-miniTol2-opt-CRY-eGFP.

Supplementary Table 3

Driver/Effector	Transgenic animals	Number of F0 founder (adult)
Driver line	*Tg*(*ubb*:*QFDBD-2xAD*-VP16-EcR*, *α-cry*:*EGFP*)	41
	Tg(*ubb*:*QFDBD-2xAD*-VP16*, *α-cry*:*EGFP*)	35
	Tg(*ubb*:*QFDBD-AD*-MD-AD-EcR*, *α-cry*:*EGFP*)*	22
	Tg(*ubb*:*QFDBD-AD-EcR*, *α-cry*:*EGFP*)*	28
	Tg(*elavl3*:*QFDBD-2xAD*-VP16*, *α-cry*:*EGFP*)	30
	Tg(*elavl3*:*QFDBD-2xAD*-VP16-EcR*, *α-cry*:*EGFP*)	30
	Tg(*ubb*:*Gal4DBD-VP16*-EcR*, *α-cry*:*EGFP*)	31
Effector line	*Tg*(*5xQUAS*:*ZsGreen-P2A*, *α-cry*:*mCherry*)	29
	Tg(*9xQUAS*:*ZsGreen-P2A*, *α-cry*:*mCherry*)	23
	Tg(*13xQUAS*:*ZsGreen-P2A*, *α-cry*:*mCherry*)	26
	Tg(*5xQUAS*:*ZsGreen-P2A-*$BRAF^{V600E}$, *α-cry*:*mCherry*)	36
	Tg(*5xQUAS*:*ZsGreen-P2A-*$laminA^{\Delta 37}$, *α-cry*:*EGFP*)	31
	Tg(*10xUAS*:*ZsGreen-P2A-*$braf^{V610E}$, *α-cry*:*EGFP*)	30

Supplementary Table 3. Transgenic lines generated in this study. Asterisk (*) indicates transgenic driver lines with gross embryonic abnormality only when exposed to Teb.

Supplementary Table 4

Laser		Detector			
Name (nm)	Intensity (%)	Name	Type	Gain	Offset
Diode 405 (405nm)	15.0034%	PMT1	PMT (410nm - 495nm)	900	0
Argon (488nm)	2.0001%	PMT2	PMT (495nm - 592nm)	1,050	0
HeNe 633 (633nm)	2.0001%	HyD3	HyD (592nm - 751nm) Standard mode	50	-0.01

Supplementary Table 4. Laser and detector information for the imaging of cells. Fluorescence images were acquired using a confocal laser scanning microscopy (TCS-SP8, LAS X; Leica), with constant excitation, emission, pinhole, and exposure time parameters, such as; 1. Objective lens: HC PL APO CS2 40x/1.10 WATER, 2. Zoom: 2.5x, 3. Pinhole: 77.2μm (1AU), 4. Line Average: 6.
