# Supplementary material for: IQ-Switch is a QF-based innocuous, silencing-free, and inducible gene switch system in zebrafish
Source: Commun Biol. 2021 Dec 16;4:1405. doi: 10.1038/s42003-021-02923-3 (PMC8677817; doi:10.1038/s42003-021-02923-3)
Supplement: Supplementary file 3 — Supplementary data 2 [file 42003_2021_2923_MOESM3_ESM.docx]

p5xQUAS-ZsGreen-P2A, α-cry:mCherry ctaaattgtaagcgttaatattttgttaaaattcgcgttaaatttttgttaaatcagctcattttttaaccaataggccgaaatcggcaaaatcccttataaatcaaaagaatagaccgagatagggttgagtgttgttccagtttggaacaagagtccactattaaagaacgtggactccaacgtcaaagggcgaaaaaccgtctatcagggcgatggcccactacgtgaaccatcaccctaatcaagttttttggggtcgaggtgccgtaaagcactaaatcggaaccctaaagggagcccccgatttagagcttgacggggaaagccggcgaacgtggcgagaaaggaagggaagaaagcgaaaggagcgggcgctagggcgctggcaagtgtagcggtcacgctgcgcgtaaccaccacacccgccgcgcttaatgcgccgctacagggcgcgtcccattcgccattcaggctgcgcaactgttgggaagggcgatcggtgcgggcctcttcgctattacgccagctggcgaaagggggatgtgctgcaaggcgattaagttgggtaacgccagggttttcccagtcacgacgttgtaaaacgacggccagtgagcgcgcgtaatacgactcactatagggcgaattgGGTACCCAGAGGTGTAAAAAGTACTCAAAAATTTTACTCAAGTGAAAGTACAAGTACTTAGGGAAAATTTTACTCAATTAAAAGTAAAAGTATCTGGCTAGAATCTTACTTGAGTAAAAGTAAAAAAGTACTCCATTAAAATTGTACTTGAGTATTCTCGAGgtcgaCAACTTTGTATAGAAAAGTTGGGGTAATCGCTTATCCTCGGATAAACAATTATCCTCACGGGTAATCGCTTATCCGCTCGGGTAATCGCTTATCCTCGGGTAATCGCTTATCCTTGCAAGGGTCGACTCTAGAGGGTATATAATGGATCCCATCGCGTCTCAGCCTCACTTTGAGCTCCTCCACACGAATTCCCTCGACCTCGAAGACGCGTCAAGTtTGTACAAAAAAGCAGGTCACCATGGATCTGTACGACGATGACGATAAGGCCCAGTCCAAGCACGGCCTGACCAAGGAGATGACCATGAAGTACCGCATGGAGGGCTGCGTGGACGGCCACAAGTTCGTGATCACCGGCGAGGGCATCGGCTACCCCTTCAAGGGCAAGCAGGCCATCAACCTGTGCGTGGTGGAGGGCGGCCCCTTGCCCTTCGCCGAGGACATCTTGTCCGCCGCCTTCATGTACGGCAACCGCGTGTTCACCGAGTACCCCCAGGACATCGTCGACTACTTCAAGAACTCCTGCCCCGCCGGCTACACCTGGGACCGCTCCTTCCTGTTCGAGGACGGCGCCGTGTGCATCTGCAACGCCGACATCACCGTGAGCGTGGAGGAGAACTGCATGTACCACGAGTCCAAGTTCTACGGCGTGAACTTCCCCGCCGACGGCCCCGTGATGAAGAAGATGACCGACAACTGGGAGCCCTCCTGCGAGAAGATCATCCCCGTGCCCAAGCAGGGCATCTTGAAGGGCGACGTGAGCATGTACCTGCTGCTGAAGGACGGTGGCCGCTTGCGCTGCCAGTTCGACACCGTGTACAAGGCCAAGTCCGTGCCCCGCAAGATGCCCGACTGGCACTTCATCCAGCACAAGCTGACCCGCGAGGACCGCAGCGACGCCAAGAACCAGAAGTGGCACCTGACCGAGCACGCCATCGCCTCCGGCTCCGCCTTGCCCGCATGCGGAAGCGGAGCTACTAACTTCAGCCTGCTGAAGCAGGCTGGAGACGTGGAGGAGAACCCTGGACCTagatCTCGAGgtcgacggtatcgataagcttgatatcgaattcctgcagcccgggggatccactagtaattaagtctcagccaccgttaactgaacatgtcaaaacctgtggagactgttgagatttgatgttctgaaaagataaagcctataaataaaatgttgcccaaatttcctgcctgatgtttttctttgtctttgctacatggctttgctgctcggatcggctcactctgtgtatgccacgttcactttgtactctccttctcacggtaggtttattatttttagatgtgcagttagtttctgtgaaataacacaccacacactgatattgtctgtgcattgacttggtgagtgcacattgtttttgatcttgacatatttatatttgattgatcaggtgaactgtgtgaatctaaagtgctccatacagatgttctgcattgaaaatattctcattttattagtggaagtgagtgtatgccacatccaatcaatttcagcaaacaccccagtatgatttaatgcaaaaaaatgaaggtatcaaacacgcattactactttgcagttaaatatttaacatttattccaacacgaaaaaaagcagtaaataacactttgacaaacacgtcaggacatcttatttttgtcaccctcacaggcaatttagtataatatattatatatatatatatatcatataataatattcagtataatatatatatatatatcatattataatattcagtataatataaaacacaaacacatatatgtataatataatataacatttttatttattgagatgcctctatggaccgtgttataagaagtaaagatcaggagaagtaaacatgaagtgtaattatgaatactgatgttaaattaagctatgatgagttttcactgttaatttaccatctcaattaaatgttgatgcctccatgaccaagttaagcagatgagactgagacaactgtagaagacaagatgttcactttgctgaatatagctggcttgacagttatctatgactctataaatatatatatatttttttttttataaaatgatttatttataactatatatccatttctcagacaggtgcttcatatccctcactcccgtagctgtccatgctggatctgtccccgttgtttttaaaaagctaaataagttattaacatgactgcatccagcgagccaaacctgtctggtgtacagctaccagagaagcttgagatcctagtcaccgGCGGCCGCTAATATGGCaataaacgatcttcagagaaacttgcattggtaaatagtttcagcctatgcatatgtagttattcaaactgtaatgactatcttacagtattatatgtttataatagtttgatccaataaatccagatttatatgcttttcaacataaaattgtgaaatgcacagaatgtacatatacaatacatttcctacaataatgctctcttttctattaatagtgtgcattcagtgcaggatgttatgtatttattttctgtagtgcaaactttttcaaagaattttccagtctaagcttttcagtcaagaaaaaaaaaagcgattgattatctgtgatatttaaaaaaattagctcttaaatctggtgaaccaagtggccacaacacattaaatactgacgttctacagccatgtagcttcatctggtctggtttgttttggcaggcacttattttccggtgatagttggccacaagagtattgtctgagccattcagtgctagactgtcattctcaggtcagagtccaccccgctgattctgctgacaacactgttcccaccatgagataatgccattccagagagatccatttgtaagcccctctttctgcagcacaggtatataaccaggggtctgcctccactaaggccggcacacatcatttggggatctttgtactgtGGATCCACCGGTCGCCACCATGGTGAGCAAGGGCGAGGAGGATAACATGGCCATCATCAAGGAGTTCATGCGCTTCAAGGTGCACATGGAGGGCTCCGTGAACGGCCACGAGTTCGAGATCGAGGGCGAGGGCGAGGGCCGCCCCTACGAGGGCACCCAGACCGCCAAGCTGAAGGTGACCAAGGGTGGCCCCCTGCCCTTCGCCTGGGACATCCTGTCCCCTCAGTTCATGTACGGCTCCAAGGCCTACGTGAAGCACCCCGCCGACATCCCCGACTACTTGAAGCTGTCCTTCCCCGAGGGCTTCAAGTGGGAGCGCGTGATGAACTTCGAGGACGGCGGCGTGGTGACCGTGACCCAGGACTCCTCCCTGCAGGACGGCGAGTTCATCTACAAGGTGAAGCTGCGCGGCACCAACTTCCCCTCCGACGGCCCCGTAATGCAGAAGAAGACCATGGGCTGGGAGGCCTCCTCCGAGCGGATGTACCCCGAGGACGGCGCCCTGAAGGGCGAGATCAAGCAGAGGCTGAAGCTGAAGGACGGCGGCCACTACGACGCTGAGGTCAAGACCACCTACAAGGCCAAGAAGCCCGTGCAGCTGCCCGGCGCCTACAACGTCAACATCAAGTTGGACATCACCTCCCACAACGAGGACTACACCATCGTGGAACAGTACGAACGCGCCGAGGGCCGCCACTCCACCGGCGGCATGGACGAGCTGTACAAGTAAAGCGGCCGCGACTCTAGATCATAATCAGCCATACCACATTTGTAGAGGTTTTACTTGCTTTAAAAAACCTCCCACACCTCCCCCTGAACCTGAAACATAAAATGAATGCAATTGTTGTTGTTAACTTGTTTATTGCAGCTTATAATGGTTACAAATAAAGCAATAGCATCACAAATTTCACAAATAAAGCATTTTTTTCACTGCATTCTAGTTGTGGTTTGTCCAAACTCATCAATGtatctaagccgcggtgGAGCTCAAGTGATCTCCAAAAAATAAGTACTTTTTGACTGTAAATAAAATTGTAAGGAGTAAAAAGTACTTTTTTTTCTAAAAAAATGTAATTAAGTAAAAGTAAAAGTATTGATTTTTAATTGTACTCAAGTAAAGTAAAAATCCCCAAAAATAATACTTAAGTACAGTAATCAAGTAAAATTACTCAAGTACTTTACACCGAGCTCcagcttttgttccctttagtgagggttaattgcgcgcttggcgtaatcatggtcatagctgtttcctgtgtgaaattgttatccgctcacaattccacacaacatacgagccggaagcataaagtgtaaagcctggggtgcctaatgagtgagctaactcacattaattgcgttgcgctcactgcccgctttccagtcgggaaacctgtcgtgccagctgcattaatgaatcggccaacgcgcggggagaggcggtttgcgtattgggcgctcttccgcttcctcgctcactgactcgctgcgctcggtcgttcggctgcggcgagcggtatcagctcactcaaaggcggtaatacggttatccacagaatcaggggataacgcaggaaagaacatgtgagcaaaaggccagcaaaaggccaggaaccgtaaaaaggccgcgttgctggcgtttttccataggctccgcccccctgacgagcatcacaaaaatcgacgctcaagtcagaggtggcgaaacccgacaggactataaagataccaggcgtttccccctggaagctccctcgtgcgctctcctgttccgaccctgccgcttaccggatacctgtccgcctttctcccttcgggaagcgtggcgctttctcatagctcacgctgtaggtatctcagttcggtgtaggtcgttcgctccaagctgggctgtgtgcacgaaccccccgttcagcccgaccgctgcgccttatccggtaactatcgtcttgagtccaacccggtaagacacgacttatcgccactggcagcagccactggtaacaggattagcagagcgaggtatgtaggcggtgctacagagttcttgaagtggtggcctaactacggctacactagaaggacagtatttggtatctgcgctctgctgaagccagttaccttcggaaaaagagttggtagctcttgatccggcaaacaaaccaccgctggtagcggtggtttttttgtttgcaagcagcagattacgcgcagaaaaaaaggatctcaagaagatcctttgatcttttctacggggtctgacgctcagtggaacgaaaactcacgttaagggattttggtcatgagattatcaaaaaggatcttcacctagatccttttaaattaaaaatgaagttttaaatcaatctaaagtatatatgagtaaacttggtctgacagttaccaatgcttaatcagtgaggcacctatctcagcgatctgtctatttcgttcatccatagttgcctgactccccgtcgtgtagataactacgatacgggagggcttaccatctggccccagtgctgcaatgataccgcgagacccacgctcaccggctccagatttatcagcaataaaccagccagccggaagggccgagcgcagaagtggtcctgcaactttatccgcctccatccagtctattaattgttgccgggaagctagagtaagtagttcgccagttaatagtttgcgcaacgttgttgccattgctacaggcatcgtggtgtcacgctcgtcgtttggtatggcttcattcagctccggttcccaacgatcaaggcgagttacatgatcccccatgttgtgcaaaaaagcggttagctccttcggtcctccgatcgttgtcagaagtaagttggccgcagtgttatcactcatggttatggcagcactgcataattctcttactgtcatgccatccgtaagatgcttttctgtgactggtgagtactcaaccaagtcattctgagaatagtgtatgcggcgaccgagttgctcttgcccggcgtcaatacgggataataccgcgccacatagcagaactttaaaagtgctcatcattggaaaacgttcttcggggcgaaaactctcaaggatcttaccgctgttgagatccagttcgatgtaacccactcgtgcacccaactgatcttcagcatcttttactttcaccagcgtttctgggtgagcaaaaacaggaaggcaaaatgccgcaaaaaagggaataagggcgacacggaaatgttgaatactcatactcttcctttttcaatattattgaagcatttatcagggttattgtctcatgagcggatacatatttgaatgtatttagaaaaataaacaaataggggttccgcgcacatttccccgaaaagtgccac

p9xQUAS-ZsGreen-P2A, α-cry:mCherry ctaaattgtaagcgttaatattttgttaaaattcgcgttaaatttttgttaaatcagctcattttttaaccaataggccgaaatcggcaaaatcccttataaatcaaaagaatagaccgagatagggttgagtgttgttccagtttggaacaagagtccactattaaagaacgtggactccaacgtcaaagggcgaaaaaccgtctatcagggcgatggcccactacgtgaaccatcaccctaatcaagttttttggggtcgaggtgccgtaaagcactaaatcggaaccctaaagggagcccccgatttagagcttgacggggaaagccggcgaacgtggcgagaaaggaagggaagaaagcgaaaggagcgggcgctagggcgctggcaagtgtagcggtcacgctgcgcgtaaccaccacacccgccgcgcttaatgcgccgctacagggcgcgtcccattcgccattcaggctgcgcaactgttgggaagggcgatcggtgcgggcctcttcgctattacgccagctggcgaaagggggatgtgctgcaaggcgattaagttgggtaacgccagggttttcccagtcacgacgttgtaaaacgacggccagtgagcgcgcgtaatacgactcactatagggcgaattgGGTACCCAGAGGTGTAAAAAGTACTCAAAAATTTTACTCAAGTGAAAGTACAAGTACTTAGGGAAAATTTTACTCAATTAAAAGTAAAAGTATCTGGCTAGAATCTTACTTGAGTAAAAGTAAAAAAGTACTCCATTAAAATTGTACTTGAGTATTCTCGAGgtcgaCAACTTTGTATAGAAAAGTTGGGGTAATCGCTTATCCTCGGATAAACAATTATCCTCACGGGTAATCGCTTATCCGCTCGGGTAATCGCTTATCCTCTCGGGTAATCGCTTATCCTCGGATAAACAATTATCCTCACGGGTAATCGCTTATCCGCTCGGGTAATCGCTTATCCTCGGGTAATCGCTTATCCTTGCAAGGGTCGACTCTAGAGGGTATATAATGGATCCCATCGCGTCTCAGCCTCACTTTGAGCTCCTCCACACGAATTCCCTCGACCTCGAAGACGCGTCAAGTtTGTACAAAAAAGCAGGTCACCATGGATCTGTACGACGATGACGATAAGGCCCAGTCCAAGCACGGCCTGACCAAGGAGATGACCATGAAGTACCGCATGGAGGGCTGCGTGGACGGCCACAAGTTCGTGATCACCGGCGAGGGCATCGGCTACCCCTTCAAGGGCAAGCAGGCCATCAACCTGTGCGTGGTGGAGGGCGGCCCCTTGCCCTTCGCCGAGGACATCTTGTCCGCCGCCTTCATGTACGGCAACCGCGTGTTCACCGAGTACCCCCAGGACATCGTCGACTACTTCAAGAACTCCTGCCCCGCCGGCTACACCTGGGACCGCTCCTTCCTGTTCGAGGACGGCGCCGTGTGCATCTGCAACGCCGACATCACCGTGAGCGTGGAGGAGAACTGCATGTACCACGAGTCCAAGTTCTACGGCGTGAACTTCCCCGCCGACGGCCCCGTGATGAAGAAGATGACCGACAACTGGGAGCCCTCCTGCGAGAAGATCATCCCCGTGCCCAAGCAGGGCATCTTGAAGGGCGACGTGAGCATGTACCTGCTGCTGAAGGACGGTGGCCGCTTGCGCTGCCAGTTCGACACCGTGTACAAGGCCAAGTCCGTGCCCCGCAAGATGCCCGACTGGCACTTCATCCAGCACAAGCTGACCCGCGAGGACCGCAGCGACGCCAAGAACCAGAAGTGGCACCTGACCGAGCACGCCATCGCCTCCGGCTCCGCCTTGCCCGCATGCGGAAGCGGAGCTACTAACTTCAGCCTGCTGAAGCAGGCTGGAGACGTGGAGGAGAACCCTGGACCTagatCTCGAGgtcgacggtatcgataagcttgatatcgaattcctgcagcccgggggatccactagtaattaagtctcagccaccgttaactgaacatgtcaaaacctgtggagactgttgagatttgatgttctgaaaagataaagcctataaataaaatgttgcccaaatttcctgcctgatgtttttctttgtctttgctacatggctttgctgctcggatcggctcactctgtgtatgccacgttcactttgtactctccttctcacggtaggtttattatttttagatgtgcagttagtttctgtgaaataacacaccacacactgatattgtctgtgcattgacttggtgagtgcacattgtttttgatcttgacatatttatatttgattgatcaggtgaactgtgtgaatctaaagtgctccatacagatgttctgcattgaaaatattctcattttattagtggaagtgagtgtatgccacatccaatcaatttcagcaaacaccccagtatgatttaatgcaaaaaaatgaaggtatcaaacacgcattactactttgcagttaaatatttaacatttattccaacacgaaaaaaagcagtaaataacactttgacaaacacgtcaggacatcttatttttgtcaccctcacaggcaatttagtataatatattatatatatatatatatcatataataatattcagtataatatatatatatatatcatattataatattcagtataatataaaacacaaacacatatatgtataatataatataacatttttatttattgagatgcctctatggaccgtgttataagaagtaaagatcaggagaagtaaacatgaagtgtaattatgaatactgatgttaaattaagctatgatgagttttcactgttaatttaccatctcaattaaatgttgatgcctccatgaccaagttaagcagatgagactgagacaactgtagaagacaagatgttcactttgctgaatatagctggcttgacagttatctatgactctataaatatatatatatttttttttttataaaatgatttatttataactatatatccatttctcagacaggtgcttcatatccctcactcccgtagctgtccatgctggatctgtccccgttgtttttaaaaagctaaataagttattaacatgactgcatccagcgagccaaacctgtctggtgtacagctaccagagaagcttgagatcctagtcaccgGCGGCCGCTAATATGGCaataaacgatcttcagagaaacttgcattggtaaatagtttcagcctatgcatatgtagttattcaaactgtaatgactatcttacagtattatatgtttataatagtttgatccaataaatccagatttatatgcttttcaacataaaattgtgaaatgcacagaatgtacatatacaatacatttcctacaataatgctctcttttctattaatagtgtgcattcagtgcaggatgttatgtatttattttctgtagtgcaaactttttcaaagaattttccagtctaagcttttcagtcaagaaaaaaaaaagcgattgattatctgtgatatttaaaaaaattagctcttaaatctggtgaaccaagtggccacaacacattaaatactgacgttctacagccatgtagcttcatctggtctggtttgttttggcaggcacttattttccggtgatagttggccacaagagtattgtctgagccattcagtgctagactgtcattctcaggtcagagtccaccccgctgattctgctgacaacactgttcccaccatgagataatgccattccagagagatccatttgtaagcccctctttctgcagcacaggtatataaccaggggtctgcctccactaaggccggcacacatcatttggggatctttgtactgtGGATCCACCGGTCGCCACCATGGTGAGCAAGGGCGAGGAGGATAACATGGCCATCATCAAGGAGTTCATGCGCTTCAAGGTGCACATGGAGGGCTCCGTGAACGGCCACGAGTTCGAGATCGAGGGCGAGGGCGAGGGCCGCCCCTACGAGGGCACCCAGACCGCCAAGCTGAAGGTGACCAAGGGTGGCCCCCTGCCCTTCGCCTGGGACATCCTGTCCCCTCAGTTCATGTACGGCTCCAAGGCCTACGTGAAGCACCCCGCCGACATCCCCGACTACTTGAAGCTGTCCTTCCCCGAGGGCTTCAAGTGGGAGCGCGTGATGAACTTCGAGGACGGCGGCGTGGTGACCGTGACCCAGGACTCCTCCCTGCAGGACGGCGAGTTCATCTACAAGGTGAAGCTGCGCGGCACCAACTTCCCCTCCGACGGCCCCGTAATGCAGAAGAAGACCATGGGCTGGGAGGCCTCCTCCGAGCGGATGTACCCCGAGGACGGCGCCCTGAAGGGCGAGATCAAGCAGAGGCTGAAGCTGAAGGACGGCGGCCACTACGACGCTGAGGTCAAGACCACCTACAAGGCCAAGAAGCCCGTGCAGCTGCCCGGCGCCTACAACGTCAACATCAAGTTGGACATCACCTCCCACAACGAGGACTACACCATCGTGGAACAGTACGAACGCGCCGAGGGCCGCCACTCCACCGGCGGCATGGACGAGCTGTACAAGTAAAGCGGCCGCGACTCTAGATCATAATCAGCCATACCACATTTGTAGAGGTTTTACTTGCTTTAAAAAACCTCCCACACCTCCCCCTGAACCTGAAACATAAAATGAATGCAATTGTTGTTGTTAACTTGTTTATTGCAGCTTATAATGGTTACAAATAAAGCAATAGCATCACAAATTTCACAAATAAAGCATTTTTTTCACTGCATTCTAGTTGTGGTTTGTCCAAACTCATCAATGtatctaagccgcggtgGAGCTCAAGTGATCTCCAAAAAATAAGTACTTTTTGACTGTAAATAAAATTGTAAGGAGTAAAAAGTACTTTTTTTTCTAAAAAAATGTAATTAAGTAAAAGTAAAAGTATTGATTTTTAATTGTACTCAAGTAAAGTAAAAATCCCCAAAAATAATACTTAAGTACAGTAATCAAGTAAAATTACTCAAGTACTTTACACCGAGCTCcagcttttgttccctttagtgagggttaattgcgcgcttggcgtaatcatggtcatagctgtttcctgtgtgaaattgttatccgctcacaattccacacaacatacgagccggaagcataaagtgtaaagcctggggtgcctaatgagtgagctaactcacattaattgcgttgcgctcactgcccgctttccagtcgggaaacctgtcgtgccagctgcattaatgaatcggccaacgcgcggggagaggcggtttgcgtattgggcgctcttccgcttcctcgctcactgactcgctgcgctcggtcgttcggctgcggcgagcggtatcagctcactcaaaggcggtaatacggttatccacagaatcaggggataacgcaggaaagaacatgtgagcaaaaggccagcaaaaggccaggaaccgtaaaaaggccgcgttgctggcgtttttccataggctccgcccccctgacgagcatcacaaaaatcgacgctcaagtcagaggtggcgaaacccgacaggactataaagataccaggcgtttccccctggaagctccctcgtgcgctctcctgttccgaccctgccgcttaccggatacctgtccgcctttctcccttcgggaagcgtggcgctttctcatagctcacgctgtaggtatctcagttcggtgtaggtcgttcgctccaagctgggctgtgtgcacgaaccccccgttcagcccgaccgctgcgccttatccggtaactatcgtcttgagtccaacccggtaagacacgacttatcgccactggcagcagccactggtaacaggattagcagagcgaggtatgtaggcggtgctacagagttcttgaagtggtggcctaactacggctacactagaaggacagtatttggtatctgcgctctgctgaagccagttaccttcggaaaaagagttggtagctcttgatccggcaaacaaaccaccgctggtagcggtggtttttttgtttgcaagcagcagattacgcgcagaaaaaaaggatctcaagaagatcctttgatcttttctacggggtctgacgctcagtggaacgaaaactcacgttaagggattttggtcatgagattatcaaaaaggatcttcacctagatccttttaaattaaaaatgaagttttaaatcaatctaaagtatatatgagtaaacttggtctgacagttaccaatgcttaatcagtgaggcacctatctcagcgatctgtctatttcgttcatccatagttgcctgactccccgtcgtgtagataactacgatacgggagggcttaccatctggccccagtgctgcaatgataccgcgagacccacgctcaccggctccagatttatcagcaataaaccagccagccggaagggccgagcgcagaagtggtcctgcaactttatccgcctccatccagtctattaattgttgccgggaagctagagtaagtagttcgccagttaatagtttgcgcaacgttgttgccattgctacaggcatcgtggtgtcacgctcgtcgtttggtatggcttcattcagctccggttcccaacgatcaaggcgagttacatgatcccccatgttgtgcaaaaaagcggttagctccttcggtcctccgatcgttgtcagaagtaagttggccgcagtgttatcactcatggttatggcagcactgcataattctcttactgtcatgccatccgtaagatgcttttctgtgactggtgagtactcaaccaagtcattctgagaatagtgtatgcggcgaccgagttgctcttgcccggcgtcaatacgggataataccgcgccacatagcagaactttaaaagtgctcatcattggaaaacgttcttcggggcgaaaactctcaaggatcttaccgctgttgagatccagttcgatgtaacccactcgtgcacccaactgatcttcagcatcttttactttcaccagcgtttctgggtgagcaaaaacaggaaggcaaaatgccgcaaaaaagggaataagggcgacacggaaatgttgaatactcatactcttcctttttcaatattattgaagcatttatcagggttattgtctcatgagcggatacatatttgaatgtatttagaaaaataaacaaataggggttccgcgcacatttccccgaaaagtgccac

p13xQUAS-ZsGreen-P2A, α-cry:mCherry ctaaattgtaagcgttaatattttgttaaaattcgcgttaaatttttgttaaatcagctcattttttaaccaataggccgaaatcggcaaaatcccttataaatcaaaagaatagaccgagatagggttgagtgttgttccagtttggaacaagagtccactattaaagaacgtggactccaacgtcaaagggcgaaaaaccgtctatcagggcgatggcccactacgtgaaccatcaccctaatcaagttttttggggtcgaggtgccgtaaagcactaaatcggaaccctaaagggagcccccgatttagagcttgacggggaaagccggcgaacgtggcgagaaaggaagggaagaaagcgaaaggagcgggcgctagggcgctggcaagtgtagcggtcacgctgcgcgtaaccaccacacccgccgcgcttaatgcgccgctacagggcgcgtcccattcgccattcaggctgcgcaactgttgggaagggcgatcggtgcgggcctcttcgctattacgccagctggcgaaagggggatgtgctgcaaggcgattaagttgggtaacgccagggttttcccagtcacgacgttgtaaaacgacggccagtgagcgcgcgtaatacgactcactatagggcgaattgGGTACCCAGAGGTGTAAAAAGTACTCAAAAATTTTACTCAAGTGAAAGTACAAGTACTTAGGGAAAATTTTACTCAATTAAAAGTAAAAGTATCTGGCTAGAATCTTACTTGAGTAAAAGTAAAAAAGTACTCCATTAAAATTGTACTTGAGTATTCTCGAGgtcgaCAACTTTGTATAGAAAAGTTGGGGTAATCGCTTATCCTCGGATAAACAATTATCCTCACGGGTAATCGCTTATCCGCTCGGGTAATCGCTTATCCTCGGGTAATCGCTTATCCTCGGGTAATCGCTTATCCTCGGATAAACAATTATCCTCACGGGTAATCGCTTATCCGCTCGGGTAATCGCTTATCCTCGGATAAACAATTATCCTCACGGGTAATCGCTTATCCGCTCGGGTAATCGCTTATCCTCGGGTAATCGCTTATCCTTGCAAGGGTCGACTCTAGAGGGTATATAATGGATCCCATCGCGTCTCAGCCTCACTTTGAGCTCCTCCACACGAATTCCCTCGACCTCGAAGACGCGTCAAGTtTGTACAAAAAAGCAGGTCACCATGGATCTGTACGACGATGACGATAAGGCCCAGTCCAAGCACGGCCTGACCAAGGAGATGACCATGAAGTACCGCATGGAGGGCTGCGTGGACGGCCACAAGTTCGTGATCACCGGCGAGGGCATCGGCTACCCCTTCAAGGGCAAGCAGGCCATCAACCTGTGCGTGGTGGAGGGCGGCCCCTTGCCCTTCGCCGAGGACATCTTGTCCGCCGCCTTCATGTACGGCAACCGCGTGTTCACCGAGTACCCCCAGGACATCGTCGACTACTTCAAGAACTCCTGCCCCGCCGGCTACACCTGGGACCGCTCCTTCCTGTTCGAGGACGGCGCCGTGTGCATCTGCAACGCCGACATCACCGTGAGCGTGGAGGAGAACTGCATGTACCACGAGTCCAAGTTCTACGGCGTGAACTTCCCCGCCGACGGCCCCGTGATGAAGAAGATGACCGACAACTGGGAGCCCTCCTGCGAGAAGATCATCCCCGTGCCCAAGCAGGGCATCTTGAAGGGCGACGTGAGCATGTACCTGCTGCTGAAGGACGGTGGCCGCTTGCGCTGCCAGTTCGACACCGTGTACAAGGCCAAGTCCGTGCCCCGCAAGATGCCCGACTGGCACTTCATCCAGCACAAGCTGACCCGCGAGGACCGCAGCGACGCCAAGAACCAGAAGTGGCACCTGACCGAGCACGCCATCGCCTCCGGCTCCGCCTTGCCCGCATGCGGAAGCGGAGCTACTAACTTCAGCCTGCTGAAGCAGGCTGGAGACGTGGAGGAGAACCCTGGACCTagatCTCGAGgtcgacggtatcgataagcttgatatcgaattcctgcagcccgggggatccactagtaattaagtctcagccaccgttaactgaacatgtcaaaacctgtggagactgttgagatttgatgttctgaaaagataaagcctataaataaaatgttgcccaaatttcctgcctgatgtttttctttgtctttgctacatggctttgctgctcggatcggctcactctgtgtatgccacgttcactttgtactctccttctcacggtaggtttattatttttagatgtgcagttagtttctgtgaaataacacaccacacactgatattgtctgtgcattgacttggtgagtgcacattgtttttgatcttgacatatttatatttgattgatcaggtgaactgtgtgaatctaaagtgctccatacagatgttctgcattgaaaatattctcattttattagtggaagtgagtgtatgccacatccaatcaatttcagcaaacaccccagtatgatttaatgcaaaaaaatgaaggtatcaaacacgcattactactttgcagttaaatatttaacatttattccaacacgaaaaaaagcagtaaataacactttgacaaacacgtcaggacatcttatttttgtcaccctcacaggcaatttagtataatatattatatatatatatatatcatataataatattcagtataatatatatatatatatcatattataatattcagtataatataaaacacaaacacatatatgtataatataatataacatttttatttattgagatgcctctatggaccgtgttataagaagtaaagatcaggagaagtaaacatgaagtgtaattatgaatactgatgttaaattaagctatgatgagttttcactgttaatttaccatctcaattaaatgttgatgcctccatgaccaagttaagcagatgagactgagacaactgtagaagacaagatgttcactttgctgaatatagctggcttgacagttatctatgactctataaatatatatatatttttttttttataaaatgatttatttataactatatatccatttctcagacaggtgcttcatatccctcactcccgtagctgtccatgctggatctgtccccgttgtttttaaaaagctaaataagttattaacatgactgcatccagcgagccaaacctgtctggtgtacagctaccagagaagcttgagatcctagtcaccgGCGGCCGCTAATATGGCaataaacgatcttcagagaaacttgcattggtaaatagtttcagcctatgcatatgtagttattcaaactgtaatgactatcttacagtattatatgtttataatagtttgatccaataaatccagatttatatgcttttcaacataaaattgtgaaatgcacagaatgtacatatacaatacatttcctacaataatgctctcttttctattaatagtgtgcattcagtgcaggatgttatgtatttattttctgtagtgcaaactttttcaaagaattttccagtctaagcttttcagtcaagaaaaaaaaaagcgattgattatctgtgatatttaaaaaaattagctcttaaatctggtgaaccaagtggccacaacacattaaatactgacgttctacagccatgtagcttcatctggtctggtttgttttggcaggcacttattttccggtgatagttggccacaagagtattgtctgagccattcagtgctagactgtcattctcaggtcagagtccaccccgctgattctgctgacaacactgttcccaccatgagataatgccattccagagagatccatttgtaagcccctctttctgcagcacaggtatataaccaggggtctgcctccactaaggccggcacacatcatttggggatctttgtactgtGGATCCACCGGTCGCCACCATGGTGAGCAAGGGCGAGGAGGATAACATGGCCATCATCAAGGAGTTCATGCGCTTCAAGGTGCACATGGAGGGCTCCGTGAACGGCCACGAGTTCGAGATCGAGGGCGAGGGCGAGGGCCGCCCCTACGAGGGCACCCAGACCGCCAAGCTGAAGGTGACCAAGGGTGGCCCCCTGCCCTTCGCCTGGGACATCCTGTCCCCTCAGTTCATGTACGGCTCCAAGGCCTACGTGAAGCACCCCGCCGACATCCCCGACTACTTGAAGCTGTCCTTCCCCGAGGGCTTCAAGTGGGAGCGCGTGATGAACTTCGAGGACGGCGGCGTGGTGACCGTGACCCAGGACTCCTCCCTGCAGGACGGCGAGTTCATCTACAAGGTGAAGCTGCGCGGCACCAACTTCCCCTCCGACGGCCCCGTAATGCAGAAGAAGACCATGGGCTGGGAGGCCTCCTCCGAGCGGATGTACCCCGAGGACGGCGCCCTGAAGGGCGAGATCAAGCAGAGGCTGAAGCTGAAGGACGGCGGCCACTACGACGCTGAGGTCAAGACCACCTACAAGGCCAAGAAGCCCGTGCAGCTGCCCGGCGCCTACAACGTCAACATCAAGTTGGACATCACCTCCCACAACGAGGACTACACCATCGTGGAACAGTACGAACGCGCCGAGGGCCGCCACTCCACCGGCGGCATGGACGAGCTGTACAAGTAAAGCGGCCGCGACTCTAGATCATAATCAGCCATACCACATTTGTAGAGGTTTTACTTGCTTTAAAAAACCTCCCACACCTCCCCCTGAACCTGAAACATAAAATGAATGCAATTGTTGTTGTTAACTTGTTTATTGCAGCTTATAATGGTTACAAATAAAGCAATAGCATCACAAATTTCACAAATAAAGCATTTTTTTCACTGCATTCTAGTTGTGGTTTGTCCAAACTCATCAATGtatctaagccgcggtgGAGCTCAAGTGATCTCCAAAAAATAAGTACTTTTTGACTGTAAATAAAATTGTAAGGAGTAAAAAGTACTTTTTTTTCTAAAAAAATGTAATTAAGTAAAAGTAAAAGTATTGATTTTTAATTGTACTCAAGTAAAGTAAAAATCCCCAAAAATAATACTTAAGTACAGTAATCAAGTAAAATTACTCAAGTACTTTACACCGAGCTCcagcttttgttccctttagtgagggttaattgcgcgcttggcgtaatcatggtcatagctgtttcctgtgtgaaattgttatccgctcacaattccacacaacatacgagccggaagcataaagtgtaaagcctggggtgcctaatgagtgagctaactcacattaattgcgttgcgctcactgcccgctttccagtcgggaaacctgtcgtgccagctgcattaatgaatcggccaacgcgcggggagaggcggtttgcgtattgggcgctcttccgcttcctcgctcactgactcgctgcgctcggtcgttcggctgcggcgagcggtatcagctcactcaaaggcggtaatacggttatccacagaatcaggggataacgcaggaaagaacatgtgagcaaaaggccagcaaaaggccaggaaccgtaaaaaggccgcgttgctggcgtttttccataggctccgcccccctgacgagcatcacaaaaatcgacgctcaagtcagaggtggcgaaacccgacaggactataaagataccaggcgtttccccctggaagctccctcgtgcgctctcctgttccgaccctgccgcttaccggatacctgtccgcctttctcccttcgggaagcgtggcgctttctcatagctcacgctgtaggtatctcagttcggtgtaggtcgttcgctccaagctgggctgtgtgcacgaaccccccgttcagcccgaccgctgcgccttatccggtaactatcgtcttgagtccaacccggtaagacacgacttatcgccactggcagcagccactggtaacaggattagcagagcgaggtatgtaggcggtgctacagagttcttgaagtggtggcctaactacggctacactagaaggacagtatttggtatctgcgctctgctgaagccagttaccttcggaaaaagagttggtagctcttgatccggcaaacaaaccaccgctggtagcggtggtttttttgtttgcaagcagcagattacgcgcagaaaaaaaggatctcaagaagatcctttgatcttttctacggggtctgacgctcagtggaacgaaaactcacgttaagggattttggtcatgagattatcaaaaaggatcttcacctagatccttttaaattaaaaatgaagttttaaatcaatctaaagtatatatgagtaaacttggtctgacagttaccaatgcttaatcagtgaggcacctatctcagcgatctgtctatttcgttcatccatagttgcctgactccccgtcgtgtagataactacgatacgggagggcttaccatctggccccagtgctgcaatgataccgcgagacccacgctcaccggctccagatttatcagcaataaaccagccagccggaagggccgagcgcagaagtggtcctgcaactttatccgcctccatccagtctattaattgttgccgggaagctagagtaagtagttcgccagttaatagtttgcgcaacgttgttgccattgctacaggcatcgtggtgtcacgctcgtcgtttggtatggcttcattcagctccggttcccaacgatcaaggcgagttacatgatcccccatgttgtgcaaaaaagcggttagctccttcggtcctccgatcgttgtcagaagtaagttggccgcagtgttatcactcatggttatggcagcactgcataattctcttactgtcatgccatccgtaagatgcttttctgtgactggtgagtactcaaccaagtcattctgagaatagtgtatgcggcgaccgagttgctcttgcccggcgtcaatacgggataataccgcgccacatagcagaactttaaaagtgctcatcattggaaaacgttcttcggggcgaaaactctcaaggatcttaccgctgttgagatccagttcgatgtaacccactcgtgcacccaactgatcttcagcatcttttactttcaccagcgtttctgggtgagcaaaaacaggaaggcaaaatgccgcaaaaaagggaataagggcgacacggaaatgttgaatactcatactcttcctttttcaatattattgaagcatttatcagggttattgtctcatgagcggatacatatttgaatgtatttagaaaaataaacaaataggggttccgcgcacatttccccgaaaagtgccac

p13xQUAS-MCS, α-cry:mCherry ctaaattgtaagcgttaatattttgttaaaattcgcgttaaatttttgttaaatcagctcattttttaaccaataggccgaaatcggcaaaatcccttataaatcaaaagaatagaccgagatagggttgagtgttgttccagtttggaacaagagtccactattaaagaacgtggactccaacgtcaaagggcgaaaaaccgtctatcagggcgatggcccactacgtgaaccatcaccctaatcaagttttttggggtcgaggtgccgtaaagcactaaatcggaaccctaaagggagcccccgatttagagcttgacggggaaagccggcgaacgtggcgagaaaggaagggaagaaagcgaaaggagcgggcgctagggcgctggcaagtgtagcggtcacgctgcgcgtaaccaccacacccgccgcgcttaatgcgccgctacagggcgcgtcccattcgccattcaggctgcgcaactgttgggaagggcgatcggtgcgggcctcttcgctattacgccagctggcgaaagggggatgtgctgcaaggcgattaagttgggtaacgccagggttttcccagtcacgacgttgtaaaacgacggccagtgagcgcgcgtaatacgactcactatagggcgaattgGGTACCCAGAGGTGTAAAAAGTACTCAAAAATTTTACTCAAGTGAAAGTACAAGTACTTAGGGAAAATTTTACTCAATTAAAAGTAAAAGTATCTGGCTAGAATCTTACTTGAGTAAAAGTAAAAAAGTACTCCATTAAAATTGTACTTGAGTATTCTCGAGgtcgaCAACTTTGTATAGAAAAGTTGGGGTAATCGCTTATCCTCGGATAAACAATTATCCTCACGGGTAATCGCTTATCCGCTCGGGTAATCGCTTATCCTCGGGTAATCGCTTATCCTCGGGTAATCGCTTATCCTCGGATAAACAATTATCCTCACGGGTAATCGCTTATCCGCTCGGGTAATCGCTTATCCTCGGATAAACAATTATCCTCACGGGTAATCGCTTATCCGCTCGGGTAATCGCTTATCCTCGGGTAATCGCTTATCCTTGCAAGGGTCGACTCTAGAGGGTATATAATGGATCCCATCGCGTCTCAGCCTCACTTTGAGCTCCTCCACACGAATTCCCTCGACCTCGAAGACGCGTGCATCGCTAGCGGCAGATCTCGAGGTCGACggtatcgataagcttgatatcgaattcctgcagcccgggggatccactagtaattaagtctcagccaccgttaactgaacatgtcaaaacctgtggagactgttgagatttgatgttctgaaaagataaagcctataaataaaatgttgcccaaatttcctgcctgatgtttttctttgtctttgctacatggctttgctgctcggatcggctcactctgtgtatgccacgttcactttgtactctccttctcacggtaggtttattatttttagatgtgcagttagtttctgtgaaataacacaccacacactgatattgtctgtgcattgacttggtgagtgcacattgtttttgatcttgacatatttatatttgattgatcaggtgaactgtgtgaatctaaagtgctccatacagatgttctgcattgaaaatattctcattttattagtggaagtgagtgtatgccacatccaatcaatttcagcaaacaccccagtatgatttaatgcaaaaaaatgaaggtatcaaacacgcattactactttgcagttaaatatttaacatttattccaacacgaaaaaaagcagtaaataacactttgacaaacacgtcaggacatcttatttttgtcaccctcacaggcaatttagtataatatattatatatatatatatatcatataataatattcagtataatatatatatatatatcatattataatattcagtataatataaaacacaaacacatatatgtataatataatataacatttttatttattgagatgcctctatggaccgtgttataagaagtaaagatcaggagaagtaaacatgaagtgtaattatgaatactgatgttaaattaagctatgatgagttttcactgttaatttaccatctcaattaaatgttgatgcctccatgaccaagttaagcagatgagactgagacaactgtagaagacaagatgttcactttgctgaatatagctggcttgacagttatctatgactctataaatatatatatatttttttttttataaaatgatttatttataactatatatccatttctcagacaggtgcttcatatccctcactcccgtagctgtccatgctggatctgtccccgttgtttttaaaaagctaaataagttattaacatgactgcatccagcgagccaaacctgtctggtgtacagctaccagagaagcttgagatcctagtcaccgGCGGCCGCTAATATGGCaataaacgatcttcagagaaacttgcattggtaaatagtttcagcctatgcatatgtagttattcaaactgtaatgactatcttacagtattatatgtttataatagtttgatccaataaatccagatttatatgcttttcaacataaaattgtgaaatgcacagaatgtacatatacaatacatttcctacaataatgctctcttttctattaatagtgtgcattcagtgcaggatgttatgtatttattttctgtagtgcaaactttttcaaagaattttccagtctaagcttttcagtcaagaaaaaaaaaagcgattgattatctgtgatatttaaaaaaattagctcttaaatctggtgaaccaagtggccacaacacattaaatactgacgttctacagccatgtagcttcatctggtctggtttgttttggcaggcacttattttccggtgatagttggccacaagagtattgtctgagccattcagtgctagactgtcattctcaggtcagagtccaccccgctgattctgctgacaacactgttcccaccatgagataatgccattccagagagatccatttgtaagcccctctttctgcagcacaggtatataaccaggggtctgcctccactaaggccggcacacatcatttggggatctttgtactgtGGATCCACCGGTCGCCACCATGGTGAGCAAGGGCGAGGAGGATAACATGGCCATCATCAAGGAGTTCATGCGCTTCAAGGTGCACATGGAGGGCTCCGTGAACGGCCACGAGTTCGAGATCGAGGGCGAGGGCGAGGGCCGCCCCTACGAGGGCACCCAGACCGCCAAGCTGAAGGTGACCAAGGGTGGCCCCCTGCCCTTCGCCTGGGACATCCTGTCCCCTCAGTTCATGTACGGCTCCAAGGCCTACGTGAAGCACCCCGCCGACATCCCCGACTACTTGAAGCTGTCCTTCCCCGAGGGCTTCAAGTGGGAGCGCGTGATGAACTTCGAGGACGGCGGCGTGGTGACCGTGACCCAGGACTCCTCCCTGCAGGACGGCGAGTTCATCTACAAGGTGAAGCTGCGCGGCACCAACTTCCCCTCCGACGGCCCCGTAATGCAGAAGAAGACCATGGGCTGGGAGGCCTCCTCCGAGCGGATGTACCCCGAGGACGGCGCCCTGAAGGGCGAGATCAAGCAGAGGCTGAAGCTGAAGGACGGCGGCCACTACGACGCTGAGGTCAAGACCACCTACAAGGCCAAGAAGCCCGTGCAGCTGCCCGGCGCCTACAACGTCAACATCAAGTTGGACATCACCTCCCACAACGAGGACTACACCATCGTGGAACAGTACGAACGCGCCGAGGGCCGCCACTCCACCGGCGGCATGGACGAGCTGTACAAGTAAAGCGGCCGCGACTCTAGATCATAATCAGCCATACCACATTTGTAGAGGTTTTACTTGCTTTAAAAAACCTCCCACACCTCCCCCTGAACCTGAAACATAAAATGAATGCAATTGTTGTTGTTAACTTGTTTATTGCAGCTTATAATGGTTACAAATAAAGCAATAGCATCACAAATTTCACAAATAAAGCATTTTTTTCACTGCATTCTAGTTGTGGTTTGTCCAAACTCATCAATGtatctaagccgcggtgGAGCTCAAGTGATCTCCAAAAAATAAGTACTTTTTGACTGTAAATAAAATTGTAAGGAGTAAAAAGTACTTTTTTTTCTAAAAAAATGTAATTAAGTAAAAGTAAAAGTATTGATTTTTAATTGTACTCAAGTAAAGTAAAAATCCCCAAAAATAATACTTAAGTACAGTAATCAAGTAAAATTACTCAAGTACTTTACACCGAGCTCcagcttttgttccctttagtgagggttaattgcgcgcttggcgtaatcatggtcatagctgtttcctgtgtgaaattgttatccgctcacaattccacacaacatacgagccggaagcataaagtgtaaagcctggggtgcctaatgagtgagctaactcacattaattgcgttgcgctcactgcccgctttccagtcgggaaacctgtcgtgccagctgcattaatgaatcggccaacgcgcggggagaggcggtttgcgtattgggcgctcttccgcttcctcgctcactgactcgctgcgctcggtcgttcggctgcggcgagcggtatcagctcactcaaaggcggtaatacggttatccacagaatcaggggataacgcaggaaagaacatgtgagcaaaaggccagcaaaaggccaggaaccgtaaaaaggccgcgttgctggcgtttttccataggctccgcccccctgacgagcatcacaaaaatcgacgctcaagtcagaggtggcgaaacccgacaggactataaagataccaggcgtttccccctggaagctccctcgtgcgctctcctgttccgaccctgccgcttaccggatacctgtccgcctttctcccttcgggaagcgtggcgctttctcatagctcacgctgtaggtatctcagttcggtgtaggtcgttcgctccaagctgggctgtgtgcacgaaccccccgttcagcccgaccgctgcgccttatccggtaactatcgtcttgagtccaacccggtaagacacgacttatcgccactggcagcagccactggtaacaggattagcagagcgaggtatgtaggcggtgctacagagttcttgaagtggtggcctaactacggctacactagaaggacagtatttggtatctgcgctctgctgaagccagttaccttcggaaaaagagttggtagctcttgatccggcaaacaaaccaccgctggtagcggtggtttttttgtttgcaagcagcagattacgcgcagaaaaaaaggatctcaagaagatcctttgatcttttctacggggtctgacgctcagtggaacgaaaactcacgttaagggattttggtcatgagattatcaaaaaggatcttcacctagatccttttaaattaaaaatgaagttttaaatcaatctaaagtatatatgagtaaacttggtctgacagttaccaatgcttaatcagtgaggcacctatctcagcgatctgtctatttcgttcatccatagttgcctgactccccgtcgtgtagataactacgatacgggagggcttaccatctggccccagtgctgcaatgataccgcgagacccacgctcaccggctccagatttatcagcaataaaccagccagccggaagggccgagcgcagaagtggtcctgcaactttatccgcctccatccagtctattaattgttgccgggaagctagagtaagtagttcgccagttaatagtttgcgcaacgttgttgccattgctacaggcatcgtggtgtcacgctcgtcgtttggtatggcttcattcagctccggttcccaacgatcaaggcgagttacatgatcccccatgttgtgcaaaaaagcggttagctccttcggtcctccgatcgttgtcagaagtaagttggccgcagtgttatcactcatggttatggcagcactgcataattctcttactgtcatgccatccgtaagatgcttttctgtgactggtgagtactcaaccaagtcattctgagaatagtgtatgcggcgaccgagttgctcttgcccggcgtcaatacgggataataccgcgccacatagcagaactttaaaagtgctcatcattggaaaacgttcttcggggcgaaaactctcaaggatcttaccgctgttgagatccagttcgatgtaacccactcgtgcacccaactgatcttcagcatcttttactttcaccagcgtttctgggtgagcaaaaacaggaaggcaaaatgccgcaaaaaagggaataagggcgacacggaaatgttgaatactcatactcttcctttttcaatattattgaagcatttatcagggttattgtctcatgagcggatacatatttgaatgtatttagaaaaataaacaaataggggttccgcgcacatttccccgaaaagtgccac

p17xQUAS-Luc, α-cry:mCherry

ctaaattgtaagcgttaatattttgttaaaattcgcgttaaatttttgttaaatcagctcattttttaaccaataggccgaaatcggcaaaatcccttataaatcaaaagaatagaccgagatagggttgagtgttgttccagtttggaacaagagtccactattaaagaacgtggactccaacgtcaaagggcgaaaaaccgtctatcagggcgatggcccactacgtgaaccatcaccctaatcaagttttttggggtcgaggtgccgtaaagcactaaatcggaaccctaaagggagcccccgatttagagcttgacggggaaagccggcgaacgtggcgagaaaggaagggaagaaagcgaaaggagcgggcgctagggcgctggcaagtgtagcggtcacgctgcgcgtaaccaccacacccgccgcgcttaatgcgccgctacagggcgcgtcccattcgccattcaggctgcgcaactgttgggaagggcgatcggtgcgggcctcttcgctattacgccagctggcgaaagggggatgtgctgcaaggcgattaagttgggtaacgccagggttttcccagtcacgacgttgtaaaacgacggccagtgagcgcgcgtaatacgactcactatagggcgaattgGGTACCCAGAGGTGTAAAAAGTACTCAAAAATTTTACTCAAGTGAAAGTACAAGTACTTAGGGAAAATTTTACTCAATTAAAAGTAAAAGTATCTGGCTAGAATCTTACTTGAGTAAAAGTAAAAAAGTACTCCATTAAAATTGTACTTGAGTATTCTCGAGgtcgaCAACTTTGTATAGAAAAGTTGGGGTAATCGCTTATCCTCGGATAAACAATTATCCTCACGGGTAATCGCTTATCCGCTCGGGTAATCGCTTATCCTCGGGTAATCGCTTATCCTCGGGTAATCGCTTATCCTCGGGTAATCGCTTATCCTCGGATAAACAATTATCCTCACGGGTAATCGCTTATCCGCTCGGGTAATCGCTTATCCTCGGATAAACAATTATCCTCACGGGTAATCGCTTATCCGCTCGGGTAATCGCTTATCCTCGGATAAACAATTATCCTCACGGGTAATCGCTTATCCGCTCGGGTAATCGCTTATCCTCGGGTAATCGCTTATCCTTGCAAGGGTCGACTCTAGAGGGTATATAATGGATCCCATCGCGTCTCAGCCTCACTTTGAGCTCCTCCACACGAATTCCCTCGACCTCGAAGACGCGTGCCACCatggaagacgccaaaaacataaagaaaggcccggcgccattctatccgctggaagatggaaccgctggagagcaactgcataaggctatgaagagatacgccctggttcctggaacaattgcttttacagatgcacatatcgaggtggacatcacttacgctgagtacttcgaaatgtccgttcggttggcagaagctatgaaacgatatgggctgaatacaaatcacagaatcgtcgtatgcagtgaaaactctcttcaattctttatgccggtgttgggcgcgttatttatcggagttgcagttgcgcccgcgaacgacatttataatgaacgtgaattgctcaacagtatgggcatttcgcagcctaccgtggtgttcgtttccaaaaaggggttgcaaaaaattttgaacgtgcaaaaaaagctcccaatcatccaaaaaattattatcatggattctaaaacggattaccagggatttcagtcgatgtacacgttcgtcacatctcatctacctcccggttttaatgaatacgattttgtgccagagtccttcgatagggacaagacaattgcactgatcatgaactcctctggatctactggtctgcctaaaggtgtcgctctgcctcatagaactgcctgcgtgagattctcgcatgccagagatcctatttttggcaatcaaatcattccggatactgcgattttaagtgttgttccattccatcacggttttggaatgtttactacactcggatatttgatatgtggatttcgagtcgtcttaatgtatagatttgaagaagagctgtttctgaggagccttcaggattacaagattcaaagtgcgctgctggtgccaaccctattctccttcttcgccaaaagcactctgattgacaaatacgatttatctaatttacacgaaattgcttctggtggcgctcccctctctaaggaagtcggggaagcggttgccaagaggttccatctgccaggtatcaggcaaggatatgggctcactgagactacatcagctattctgattacacccgagggggatgataaaccgggcgcggtcggtaaagttgttccattttttgaagcgaaggttgtggatctggataccgggaaaacgctgggcgttaatcaaagaggcgaactgtgtgtgagaggtcctatgattatgtccggttatgtaaacaatccggaagcgaccaacgccttgattgacaaggatggatggctacattctggagacatagcttactgggacgaagacgaacacttcttcatcgttgaccgcctgaagtctctgattaagtacaaaggctatcaggtggctcccgctgaattggaatccatcttgctccaacaccccaacatcttcgacgcaggtgtcgcaggtcttcccgacgatgacgccggtgaacttcccgccgccgttgttgttttggagcacggaaagacgatgacggaaaaagagatcgtggattacgtcgccagtcaagtaacaaccgcgaaaaagttgcgcggaggagttgtgtttgtggacgaagtaccgaaaggtcttaccggaaaactcgacgcaagaaaaatcagagagatcctcataaaggccaagaagggcggaaagatcgccgtgtaagatCTCGAGgtcgacggtatcgataagcttgatatcgaattcctgcagcccgggggatccactagtaattaagtctcagccaccgttaactgaacatgtcaaaacctgtggagactgttgagatttgatgttctgaaaagataaagcctataaataaaatgttgcccaaatttcctgcctgatgtttttctttgtctttgctacatggctttgctgctcggatcggctcactctgtgtatgccacgttcactttgtactctccttctcacggtaggtttattatttttagatgtgcagttagtttctgtgaaataacacaccacacactgatattgtctgtgcattgacttggtgagtgcacattgtttttgatcttgacatatttatatttgattgatcaggtgaactgtgtgaatctaaagtgctccatacagatgttctgcattgaaaatattctcattttattagtggaagtgagtgtatgccacatccaatcaatttcagcaaacaccccagtatgatttaatgcaaaaaaatgaaggtatcaaacacgcattactactttgcagttaaatatttaacatttattccaacacgaaaaaaagcagtaaataacactttgacaaacacgtcaggacatcttatttttgtcaccctcacaggcaatttagtataatatattatatatatatatatatcatataataatattcagtataatatatatatatatatcatattataatattcagtataatataaaacacaaacacatatatgtataatataatataacatttttatttattgagatgcctctatggaccgtgttataagaagtaaagatcaggagaagtaaacatgaagtgtaattatgaatactgatgttaaattaagctatgatgagttttcactgttaatttaccatctcaattaaatgttgatgcctccatgaccaagttaagcagatgagactgagacaactgtagaagacaagatgttcactttgctgaatatagctggcttgacagttatctatgactctataaatatatatatatttttttttttataaaatgatttatttataactatatatccatttctcagacaggtgcttcatatccctcactcccgtagctgtccatgctggatctgtccccgttgtttttaaaaagctaaataagttattaacatgactgcatccagcgagccaaacctgtctggtgtacagctaccagagaagcttgagatcctagtcaccgGCGGCCGCTAATATGGCaataaacgatcttcagagaaacttgcattggtaaatagtttcagcctatgcatatgtagttattcaaactgtaatgactatcttacagtattatatgtttataatagtttgatccaataaatccagatttatatgcttttcaacataaaattgtgaaatgcacagaatgtacatatacaatacatttcctacaataatgctctcttttctattaatagtgtgcattcagtgcaggatgttatgtatttattttctgtagtgcaaactttttcaaagaattttccagtctaagcttttcagtcaagaaaaaaaaaagcgattgattatctgtgatatttaaaaaaattagctcttaaatctggtgaaccaagtggccacaacacattaaatactgacgttctacagccatgtagcttcatctggtctggtttgttttggcaggcacttattttccggtgatagttggccacaagagtattgtctgagccattcagtgctagactgtcattctcaggtcagagtccaccccgctgattctgctgacaacactgttcccaccatgagataatgccattccagagagatccatttgtaagcccctctttctgcagcacaggtatataaccaggggtctgcctccactaaggccggcacacatcatttggggatctttgtactgtGGATCCACCGGTCGCCACCATGGTGAGCAAGGGCGAGGAGGATAACATGGCCATCATCAAGGAGTTCATGCGCTTCAAGGTGCACATGGAGGGCTCCGTGAACGGCCACGAGTTCGAGATCGAGGGCGAGGGCGAGGGCCGCCCCTACGAGGGCACCCAGACCGCCAAGCTGAAGGTGACCAAGGGTGGCCCCCTGCCCTTCGCCTGGGACATCCTGTCCCCTCAGTTCATGTACGGCTCCAAGGCCTACGTGAAGCACCCCGCCGACATCCCCGACTACTTGAAGCTGTCCTTCCCCGAGGGCTTCAAGTGGGAGCGCGTGATGAACTTCGAGGACGGCGGCGTGGTGACCGTGACCCAGGACTCCTCCCTGCAGGACGGCGAGTTCATCTACAAGGTGAAGCTGCGCGGCACCAACTTCCCCTCCGACGGCCCCGTAATGCAGAAGAAGACCATGGGCTGGGAGGCCTCCTCCGAGCGGATGTACCCCGAGGACGGCGCCCTGAAGGGCGAGATCAAGCAGAGGCTGAAGCTGAAGGACGGCGGCCACTACGACGCTGAGGTCAAGACCACCTACAAGGCCAAGAAGCCCGTGCAGCTGCCCGGCGCCTACAACGTCAACATCAAGTTGGACATCACCTCCCACAACGAGGACTACACCATCGTGGAACAGTACGAACGCGCCGAGGGCCGCCACTCCACCGGCGGCATGGACGAGCTGTACAAGTAAAGCGGCCGCGACTCTAGATCATAATCAGCCATACCACATTTGTAGAGGTTTTACTTGCTTTAAAAAACCTCCCACACCTCCCCCTGAACCTGAAACATAAAATGAATGCAATTGTTGTTGTTAACTTGTTTATTGCAGCTTATAATGGTTACAAATAAAGCAATAGCATCACAAATTTCACAAATAAAGCATTTTTTTCACTGCATTCTAGTTGTGGTTTGTCCAAACTCATCAATGtatctaagccgcggtgGAGCTCAAGTGATCTCCAAAAAATAAGTACTTTTTGACTGTAAATAAAATTGTAAGGAGTAAAAAGTACTTTTTTTTCTAAAAAAATGTAATTAAGTAAAAGTAAAAGTATTGATTTTTAATTGTACTCAAGTAAAGTAAAAATCCCCAAAAATAATACTTAAGTACAGTAATCAAGTAAAATTACTCAAGTACTTTACACCGAGCTCcagcttttgttccctttagtgagggttaattgcgcgcttggcgtaatcatggtcatagctgtttcctgtgtgaaattgttatccgctcacaattccacacaacatacgagccggaagcataaagtgtaaagcctggggtgcctaatgagtgagctaactcacattaattgcgttgcgctcactgcccgctttccagtcgggaaacctgtcgtgccagctgcattaatgaatcggccaacgcgcggggagaggcggtttgcgtattgggcgctcttccgcttcctcgctcactgactcgctgcgctcggtcgttcggctgcggcgagcggtatcagctcactcaaaggcggtaatacggttatccacagaatcaggggataacgcaggaaagaacatgtgagcaaaaggccagcaaaaggccaggaaccgtaaaaaggccgcgttgctggcgtttttccataggctccgcccccctgacgagcatcacaaaaatcgacgctcaagtcagaggtggcgaaacccgacaggactataaagataccaggcgtttccccctggaagctccctcgtgcgctctcctgttccgaccctgccgcttaccggatacctgtccgcctttctcccttcgggaagcgtggcgctttctcatagctcacgctgtaggtatctcagttcggtgtaggtcgttcgctccaagctgggctgtgtgcacgaaccccccgttcagcccgaccgctgcgccttatccggtaactatcgtcttgagtccaacccggtaagacacgacttatcgccactggcagcagccactggtaacaggattagcagagcgaggtatgtaggcggtgctacagagttcttgaagtggtggcctaactacggctacactagaaggacagtatttggtatctgcgctctgctgaagccagttaccttcggaaaaagagttggtagctcttgatccggcaaacaaaccaccgctggtagcggtggtttttttgtttgcaagcagcagattacgcgcagaaaaaaaggatctcaagaagatcctttgatcttttctacggggtctgacgctcagtggaacgaaaactcacgttaagggattttggtcatgagattatcaaaaaggatcttcacctagatccttttaaattaaaaatgaagttttaaatcaatctaaagtatatatgagtaaacttggtctgacagttaccaatgcttaatcagtgaggcacctatctcagcgatctgtctatttcgttcatccatagttgcctgactccccgtcgtgtagataactacgatacgggagggcttaccatctggccccagtgctgcaatgataccgcgagacccacgctcaccggctccagatttatcagcaataaaccagccagccggaagggccgagcgcagaagtggtcctgcaactttatccgcctccatccagtctattaattgttgccgggaagctagagtaagtagttcgccagttaatagtttgcgcaacgttgttgccattgctacaggcatcgtggtgtcacgctcgtcgtttggtatggcttcattcagctccggttcccaacgatcaaggcgagttacatgatcccccatgttgtgcaaaaaagcggttagctccttcggtcctccgatcgttgtcagaagtaagttggccgcagtgttatcactcatggttatggcagcactgcataattctcttactgtcatgccatccgtaagatgcttttctgtgactggtgagtactcaaccaagtcattctgagaatagtgtatgcggcgaccgagttgctcttgcccggcgtcaatacgggataataccgcgccacatagcagaactttaaaagtgctcatcattggaaaacgttcttcggggcgaaaactctcaaggatcttaccgctgttgagatccagttcgatgtaacccactcgtgcacccaactgatcttcagcatcttttactttcaccagcgtttctgggtgagcaaaaacaggaaggcaaaatgccgcaaaaaagggaataagggcgacacggaaatgttgaatactcatactcttcctttttcaatattattgaagcatttatcagggttattgtctcatgagcggatacatatttgaatgtatttagaaaaataaacaaataggggttccgcgcacatttccccgaaaagtgccac

pQFDBD-2x AD*-VP16*, α-cry:EGFP

ctaaattgtaagcgttaatattttgttaaaattcgcgttaaatttttgttaaatcagctcattttttaaccaataggccgaaatcggcaaaatcccttataaatcaaaagaatagaccgagatagggttgagtgttgttccagtttggaacaagagtccactattaaagaacgtggactccaacgtcaaagggcgaaaaaccgtctatcagggcgatggcccactacgtgaaccatcaccctaatcaagttttttggggtcgaggtgccgtaaagcactaaatcggaaccctaaagggagcccccgatttagagcttgacggggaaagccggcgaacgtggcgagaaaggaagggaagaaagcgaaaggagcgggcgctagggcgctggcaagtgtagcggtcacgctgcgcgtaaccaccacacccgccgcgcttaatgcgccgctacagggcgcgtcccattcgccattcaggctgcgcaactgttgggaagggcgatcggtgcgggcctcttcgctattacgccagctggcgaaagggggatgtgctgcaaggcgattaagttgggtaacgccagggttttcccagtcacgacgttgtaaaacgacggccagtgagcgcgcgtaatacgactcactatagggcgaattgGGTACCCAGAGGTGTAAAAAGTACTCAAAAATTTTACTCAAGTGAAAGTACAAGTACTTAGGGAAAATTTTACTCAATTAAAAGTAAAAGTATCTGGCTAGAATCTTACTTGAGTAAAAGTAAAAAAGTACTCCATTAAAATTGTACTTGAGTATTCTCGAGgtcgacggtatcgataagcttgatatcgaattcctgcagcccggggCCAACATGCCGCCTAAACGCAAGACACTCAATGCCGCTGCCGAAGCCAATGCCCACGCTGATGGCCATGCTGATGGCAATGCTGATGGTCATGTCGCTAACACTGCAGCAAGCAGCAACAACGCCCGTTTTGCGGACTTGACCAACATTGACACACCCGGCCTCGGCCCTACCACGACGACGTTACTTGTCGAGCCCGCTCGTTCGAAACGCCAGAGAGTCTCGAGGGCCTGTGATCAGTGTCGAGCTGCACGTGAAAAGTGTGATGGAATCCAGCCGGCTTGCTTCCCCTGTGTGTCGCAGGGCCGGTCGTGTACCTACCAGGCCAGTCCCAAGAAGCGAGGAGTCCAGACGGGCTACATCCGCACTCTCGAACTGGCTCTGGCTTGGATGTTCGAGAACGTTGCCCGCAGCGAGGACGCCCTCCACAATCTTTTGGTCCGTGATGCTGGCCAGGGCAGCGCTCTCCTGGTCGGCAAAGACTCGCCTGCTGCAGAACGCCTGCATGCAAGATGGGCGACGAGTCGAGTCAACAAAAGCATCACCCGTCTTCTCTCAGGTCAGGCCGCACAAGATCCATCTGAAGACGGCCAATCCCCGTCCGAAGACATAAATGTCCAAGATCAGGCCGCACAAGATCCATCTGAAGACGGCCAATCCCCGTCCGAAGACATAAATGTCCAAGATGAGTTCCTGGATATGGCCGACTTCGAGTTTGAGCAGATGTTTACCGATGCCCTTGGAATTGACGAGTACGGTGGGCTGCAGTGAgggggatccactagtaattaagtctcagccaccgttaactgaacatgtcaaaacctgtggagactgttgagatttgatgttctgaaaagataaagcctataaataaaatgttgcccaaatttcctgcctgatgtttttctttgtctttgctacatggctttgctgctcggatcggctcactctgtgtatgccacgttcactttgtactctccttctcacggtaggtttattatttttagatgtgcagttagtttctgtgaaataacacaccacacactgatattgtctgtgcattgacttggtgagtgcacattgtttttgatcttgacatatttatatttgattgatcaggtgaactgtgtgaatctaaagtgctccatacagatgttctgcattgaaaatattctcattttattagtggaagtgagtgtatgccacatccaatcaatttcagcaaacaccccagtatgatttaatgcaaaaaaatgaaggtatcaaacacgcattactactttgcagttaaatatttaacatttattccaacacgaaaaaaagcagtaaataacactttgacaaacacgtcaggacatcttatttttgtcaccctcacaggcaatttagtataatatattatatatatatatatatcatataataatattcagtataatatatatatatatatcatattataatattcagtataatataaaacacaaacacatatatgtataatataatataacatttttatttattgagatgcctctatggaccgtgttataagaagtaaagatcaggagaagtaaacatgaagtgtaattatgaatactgatgttaaattaagctatgatgagttttcactgttaatttaccatctcaattaaatgttgatgcctccatgaccaagttaagcagatgagactgagacaactgtagaagacaagatgttcactttgctgaatatagctggcttgacagttatctatgactctataaatatatatatatttttttttttataaaatgatttatttataactatatatccatttctcagacaggtgcttcatatccctcactcccgtagctgtccatgctggatctgtccccgttgtttttaaaaagctaaataagttattaacatgactgcatccagcgagccaaacctgtctggtgtacagctaccagagaagcttgagatcctagtcaccgGCGGCCGCTAATATGGCaataaacgatcttcagagaaacttgcattggtaaatagtttcagcctatgcatatgtagttattcaaactgtaatgactatcttacagtattatatgtttataatagtttgatccaataaatccagatttatatgcttttcaacataaaattgtgaaatgcacagaatgtacatatacaatacatttcctacaataatgctctcttttctattaatagtgtgcattcagtgcaggatgttatgtatttattttctgtagtgcaaactttttcaaagaattttccagtctaagcttttcagtcaagaaaaaaaaaagcgattgattatctgtgatatttaaaaaaattagctcttaaatctggtgaaccaagtggccacaacacattaaatactgacgttctacagccatgtagcttcatctggtctggtttgttttggcaggcacttattttccggtgatagttggccacaagagtattgtctgagccattcagtgctagactgtcattctcaggtcagagtccaccccgctgattctgctgacaacactgttcccaccatgagataatgccattccagagagatccatttgtaagcccctctttctgcagcacaggtatataaccaggggtctgcctccactaaggccggcacacatcatttggggatctttgtactgtGGATCCACCGGTCGCCACCATGGTGAGCAAGGGCGAGGAGCTGTTCACCGGGGTGGTGCCCATCCTGGTCGAGCTGGACGGCGACGTAAACGGCCACAAGTTCAGCGTGTCCGGCGAGGGCGAGGGCGATGCCACCTACGGCAAGCTGACCCTGAAGTTCATCTGCACCACCGGCAAGCTGCCCGTGCCCTGGCCCACCCTCGTGACCACCCTGACCTACGGCGTGCAGTGCTTCAGCCGCTACCCCGACCACATGAAGCAGCACGACTTCTTCAAGTCCGCCATGCCCGAAGGCTACGTCCAGGAGCGCACCATCTTCTTCAAGGACGACGGCAACTACAAGACCCGCGCCGAGGTGAAGTTCGAGGGCGACACCCTGGTGAACCGCATCGAGCTGAAGGGCATCGACTTCAAGGAGGACGGCAACATCCTGGGGCACAAGCTGGAGTACAACTACAACAGCCACAACGTCTATATCATGGCCGACAAGCAGAAGAACGGCATCAAGGTGAACTTCAAGATCCGCCACAACATCGAGGACGGCAGCGTGCAGCTCGCCGACCACTACCAGCAGAACACCCCCATCGGCGACGGCCCCGTGCTGCTGCCCGACAACCACTACCTGAGCACCCAGTCCGCCCTGAGCAAAGACCCCAACGAGAAGCGCGATCACATGGTCCTGCTGGAGTTCGTGACCGCCGCCGGGATCACTCTCGGCATGGACGAGCTGTACAAGTAAAgcggccggccgcGACTCTAGATCATAATCAGCCATACCACATTTGTAGAGGTTTTACTTGCTTTAAAAAACCTCCCACACCTCCCCCTGAACCTGAAACATAAAATGAATGCAATTGTTGTTGTTAACTTGTTTATTGCAGCTTATAATGGTTACAAATAAAGCAATAGCATCACAAATTTCACAAATAAAGCATTTTTTTCACTGCATTCTAGTTGTGGTTTGTCCAAACTCATCAATGtatctaagccgcggtgGAGCTCAAGTGATCTCCAAAAAATAAGTACTTTTTGACTGTAAATAAAATTGTAAGGAGTAAAAAGTACTTTTTTTTCTAAAAAAATGTAATTAAGTAAAAGTAAAAGTATTGATTTTTAATTGTACTCAAGTAAAGTAAAAATCCCCAAAAATAATACTTAAGTACAGTAATCAAGTAAAATTACTCAAGTACTTTACACCGAGCTCcagcttttgttccctttagtgagggttaattgcgcgcttggcgtaatcatggtcatagctgtttcctgtgtgaaattgttatccgctcacaattccacacaacatacgagccggaagcataaagtgtaaagcctggggtgcctaatgagtgagctaactcacattaattgcgttgcgctcactgcccgctttccagtcgggaaacctgtcgtgccagctgcattaatgaatcggccaacgcgcggggagaggcggtttgcgtattgggcgctcttccgcttcctcgctcactgactcgctgcgctcggtcgttcggctgcggcgagcggtatcagctcactcaaaggcggtaatacggttatccacagaatcaggggataacgcaggaaagaacatgtgagcaaaaggccagcaaaaggccaggaaccgtaaaaaggccgcgttgctggcgtttttccataggctccgcccccctgacgagcatcacaaaaatcgacgctcaagtcagaggtggcgaaacccgacaggactataaagataccaggcgtttccccctggaagctccctcgtgcgctctcctgttccgaccctgccgcttaccggatacctgtccgcctttctcccttcgggaagcgtggcgctttctcatagctcacgctgtaggtatctcagttcggtgtaggtcgttcgctccaagctgggctgtgtgcacgaaccccccgttcagcccgaccgctgcgccttatccggtaactatcgtcttgagtccaacccggtaagacacgacttatcgccactggcagcagccactggtaacaggattagcagagcgaggtatgtaggcggtgctacagagttcttgaagtggtggcctaactacggctacactagaaggacagtatttggtatctgcgctctgctgaagccagttaccttcggaaaaagagttggtagctcttgatccggcaaacaaaccaccgctggtagcggtggtttttttgtttgcaagcagcagattacgcgcagaaaaaaaggatctcaagaagatcctttgatcttttctacggggtctgacgctcagtggaacgaaaactcacgttaagggattttggtcatgagattatcaaaaaggatcttcacctagatccttttaaattaaaaatgaagttttaaatcaatctaaagtatatatgagtaaacttggtctgacagttaccaatgcttaatcagtgaggcacctatctcagcgatctgtctatttcgttcatccatagttgcctgactccccgtcgtgtagataactacgatacgggagggcttaccatctggccccagtgctgcaatgataccgcgagacccacgctcaccggctccagatttatcagcaataaaccagccagccggaagggccgagcgcagaagtggtcctgcaactttatccgcctccatccagtctattaattgttgccgggaagctagagtaagtagttcgccagttaatagtttgcgcaacgttgttgccattgctacaggcatcgtggtgtcacgctcgtcgtttggtatggcttcattcagctccggttcccaacgatcaaggcgagttacatgatcccccatgttgtgcaaaaaagcggttagctccttcggtcctccgatcgttgtcagaagtaagttggccgcagtgttatcactcatggttatggcagcactgcataattctcttactgtcatgccatccgtaagatgcttttctgtgactggtgagtactcaaccaagtcattctgagaatagtgtatgcggcgaccgagttgctcttgcccggcgtcaatacgggataataccgcgccacatagcagaactttaaaagtgctcatcattggaaaacgttcttcggggcgaaaactctcaaggatcttaccgctgttgagatccagttcgatgtaacccactcgtgcacccaactgatcttcagcatcttttactttcaccagcgtttctgggtgagcaaaaacaggaaggcaaaatgccgcaaaaaagggaataagggcgacacggaaatgttgaatactcatactcttcctttttcaatattattgaagcatttatcagggttattgtctcatgagcggatacatatttgaatgtatttagaaaaataaacaaataggggttccgcgcacatttccccgaaaagtgccac

pQFDBD-2x AD*-VP16*-EcR, α-cry:EGFP

ctaaattgtaagcgttaatattttgttaaaattcgcgttaaatttttgttaaatcagctcattttttaaccaataggccgaaatcggcaaaatcccttataaatcaaaagaatagaccgagatagggttgagtgttgttccagtttggaacaagagtccactattaaagaacgtggactccaacgtcaaagggcgaaaaaccgtctatcagggcgatggcccactacgtgaaccatcaccctaatcaagttttttggggtcgaggtgccgtaaagcactaaatcggaaccctaaagggagcccccgatttagagcttgacggggaaagccggcgaacgtggcgagaaaggaagggaagaaagcgaaaggagcgggcgctagggcgctggcaagtgtagcggtcacgctgcgcgtaaccaccacacccgccgcgcttaatgcgccgctacagggcgcgtcccattcgccattcaggctgcgcaactgttgggaagggcgatcggtgcgggcctcttcgctattacgccagctggcgaaagggggatgtgctgcaaggcgattaagttgggtaacgccagggttttcccagtcacgacgttgtaaaacgacggccagtgagcgcgcgtaatacgactcactatagggcgaattgGGTACCCAGAGGTGTAAAAAGTACTCAAAAATTTTACTCAAGTGAAAGTACAAGTACTTAGGGAAAATTTTACTCAATTAAAAGTAAAAGTATCTGGCTAGAATCTTACTTGAGTAAAAGTAAAAAAGTACTCCATTAAAATTGTACTTGAGTATTCTCGAGgtcgacggtatcgataagcttgatatcgaattcctgcagcccggggCCAACATGCCGCCTAAACGCAAGACACTCAATGCCGCTGCCGAAGCCAATGCCCACGCTGATGGCCATGCTGATGGCAATGCTGATGGTCATGTCGCTAACACTGCAGCAAGCAGCAACAACGCCCGTTTTGCGGACTTGACCAACATTGACACACCCGGCCTCGGCCCTACCACGACGACGTTACTTGTCGAGCCCGCTCGTTCGAAACGCCAGAGAGTCTCGAGGGCCTGTGATCAGTGTCGAGCTGCACGTGAAAAGTGTGATGGAATCCAGCCGGCTTGCTTCCCCTGTGTGTCGCAGGGCCGGTCGTGTACCTACCAGGCCAGTCCCAAGAAGCGAGGAGTCCAGACGGGCTACATCCGCACTCTCGAACTGGCTCTGGCTTGGATGTTCGAGAACGTTGCCCGCAGCGAGGACGCCCTCCACAATCTTTTGGTCCGTGATGCTGGCCAGGGCAGCGCTCTCCTGGTCGGCAAAGACTCGCCTGCTGCAGAACGCCTGCATGCAAGATGGGCGACGAGTCGAGTCAACAAAAGCATCACCCGTCTTCTCTCAGGTCAGGCCGCACAAGATCCATCTGAAGACGGCCAATCCCCGTCCGAAGACATAAATGTCCAAGATCAGGCCGCACAAGATCCATCTGAAGACGGCCAATCCCCGTCCGAAGACATAAATGTCCAAGATGAGTTCCTGGATATGGCCGACTTCGAGTTTGAGCAGATGTTTACCGATGCCCTTGGAATTGACGAGTACGGTGGGCTGCAGATGAGGCCTGAATGTGTCATACAGGAGCCCAGTAAAAATAAAGACAGGCAAAGACAAAAGAAAGACAAAGGAATATTATTACCTGTTAGTACGACCACAGTCGAAGACCACATGCCCCCGATCATGCAATGTGATCCACCTCCGCCCGAGGCCGCCAGGATTCACGAAGTCGTCCCGAGGTATCTTTCGGAGAAGCTGATGGAGCAGAACAGGCAGAAGAACATACCACCATTGTCGGCGAATCAGAAGTCTCTGATCGCGAGGCTCGTGTGGTACCAGGAGGGATATGAGCAGCCCTCCGACGAGGATCTCAAAAGAGTAACGCAGACTTGGCAGTCGGATGAAGAGGACGAGGAATCCGATCTACCCTTCCGCCAGATCACGGAGATGACGATCTTAACGGTCCAGTTGATCGTCGAGTTCGCCAAGGGTCTACCGGGCTTTTCGAAGATATCACAGTCTGATCAAATCACCTTATTAAAAGCCTCGTCCAGCGAGGTGATGATGCTGCGGGTGGCGAGGCGATACGACGCCGCGTCCGACAGCATTCTGTTCGCCAACAACAAGGCGTACACGCGCGACAACTACCGCAAGGCGGGCATGGCCGAGGTCATCGAAGACCTCCTACACTTCTGCCGGTGCATGTTCGCGATGGGCATGGACAATGTGCACTTTGCACTGCTCACGGCCATCGTTATATTCTCAGATCGGCCCGGGCTCGAGCAGCCGTCGCTGGTAGAAGAGATCCAGAGATACTACCTGAACACGTTGCGAATTTACATCATCAACCAGAACAGCGCGTCGTCGCGCTGCGCCGTGATCTACGGCAGGATCCTGAGCGTGCTGACCGAGCTACGCACGCTCGGCACGCAAAACTCCAACATGTGCATCTCGCTGAAGCTGAAGAACAGGAAGCTGCCGCCGTTCCTCGAGGAGATCTGGGACGTGGCGGAGGTGGCCACGACGCATCCCACGGTGCTGCCGCCCACCAACCCGGTGGTGCTATAGgggggatccactagtaattaagtctcagccaccgttaactgaacatgtcaaaacctgtggagactgttgagatttgatgttctgaaaagataaagcctataaataaaatgttgcccaaatttcctgcctgatgtttttctttgtctttgctacatggctttgctgctcggatcggctcactctgtgtatgccacgttcactttgtactctccttctcacggtaggtttattatttttagatgtgcagttagtttctgtgaaataacacaccacacactgatattgtctgtgcattgacttggtgagtgcacattgtttttgatcttgacatatttatatttgattgatcaggtgaactgtgtgaatctaaagtgctccatacagatgttctgcattgaaaatattctcattttattagtggaagtgagtgtatgccacatccaatcaatttcagcaaacaccccagtatgatttaatgcaaaaaaatgaaggtatcaaacacgcattactactttgcagttaaatatttaacatttattccaacacgaaaaaaagcagtaaataacactttgacaaacacgtcaggacatcttatttttgtcaccctcacaggcaatttagtataatatattatatatatatatatatcatataataatattcagtataatatatatatatatatcatattataatattcagtataatataaaacacaaacacatatatgtataatataatataacatttttatttattgagatgcctctatggaccgtgttataagaagtaaagatcaggagaagtaaacatgaagtgtaattatgaatactgatgttaaattaagctatgatgagttttcactgttaatttaccatctcaattaaatgttgatgcctccatgaccaagttaagcagatgagactgagacaactgtagaagacaagatgttcactttgctgaatatagctggcttgacagttatctatgactctataaatatatatatatttttttttttataaaatgatttatttataactatatatccatttctcagacaggtgcttcatatccctcactcccgtagctgtccatgctggatctgtccccgttgtttttaaaaagctaaataagttattaacatgactgcatccagcgagccaaacctgtctggtgtacagctaccagagaagcttgagatcctagtcaccgGCGGCCGCTAATATGGCaataaacgatcttcagagaaacttgcattggtaaatagtttcagcctatgcatatgtagttattcaaactgtaatgactatcttacagtattatatgtttataatagtttgatccaataaatccagatttatatgcttttcaacataaaattgtgaaatgcacagaatgtacatatacaatacatttcctacaataatgctctcttttctattaatagtgtgcattcagtgcaggatgttatgtatttattttctgtagtgcaaactttttcaaagaattttccagtctaagcttttcagtcaagaaaaaaaaaagcgattgattatctgtgatatttaaaaaaattagctcttaaatctggtgaaccaagtggccacaacacattaaatactgacgttctacagccatgtagcttcatctggtctggtttgttttggcaggcacttattttccggtgatagttggccacaagagtattgtctgagccattcagtgctagactgtcattctcaggtcagagtccaccccgctgattctgctgacaacactgttcccaccatgagataatgccattccagagagatccatttgtaagcccctctttctgcagcacaggtatataaccaggggtctgcctccactaaggccggcacacatcatttggggatctttgtactgtGGATCCACCGGTCGCCACCATGGTGAGCAAGGGCGAGGAGCTGTTCACCGGGGTGGTGCCCATCCTGGTCGAGCTGGACGGCGACGTAAACGGCCACAAGTTCAGCGTGTCCGGCGAGGGCGAGGGCGATGCCACCTACGGCAAGCTGACCCTGAAGTTCATCTGCACCACCGGCAAGCTGCCCGTGCCCTGGCCCACCCTCGTGACCACCCTGACCTACGGCGTGCAGTGCTTCAGCCGCTACCCCGACCACATGAAGCAGCACGACTTCTTCAAGTCCGCCATGCCCGAAGGCTACGTCCAGGAGCGCACCATCTTCTTCAAGGACGACGGCAACTACAAGACCCGCGCCGAGGTGAAGTTCGAGGGCGACACCCTGGTGAACCGCATCGAGCTGAAGGGCATCGACTTCAAGGAGGACGGCAACATCCTGGGGCACAAGCTGGAGTACAACTACAACAGCCACAACGTCTATATCATGGCCGACAAGCAGAAGAACGGCATCAAGGTGAACTTCAAGATCCGCCACAACATCGAGGACGGCAGCGTGCAGCTCGCCGACCACTACCAGCAGAACACCCCCATCGGCGACGGCCCCGTGCTGCTGCCCGACAACCACTACCTGAGCACCCAGTCCGCCCTGAGCAAAGACCCCAACGAGAAGCGCGATCACATGGTCCTGCTGGAGTTCGTGACCGCCGCCGGGATCACTCTCGGCATGGACGAGCTGTACAAGTAAAgcggccggccgcGACTCTAGATCATAATCAGCCATACCACATTTGTAGAGGTTTTACTTGCTTTAAAAAACCTCCCACACCTCCCCCTGAACCTGAAACATAAAATGAATGCAATTGTTGTTGTTAACTTGTTTATTGCAGCTTATAATGGTTACAAATAAAGCAATAGCATCACAAATTTCACAAATAAAGCATTTTTTTCACTGCATTCTAGTTGTGGTTTGTCCAAACTCATCAATGtatctaagccgcggtgGAGCTCAAGTGATCTCCAAAAAATAAGTACTTTTTGACTGTAAATAAAATTGTAAGGAGTAAAAAGTACTTTTTTTTCTAAAAAAATGTAATTAAGTAAAAGTAAAAGTATTGATTTTTAATTGTACTCAAGTAAAGTAAAAATCCCCAAAAATAATACTTAAGTACAGTAATCAAGTAAAATTACTCAAGTACTTTACACCGAGCTCcagcttttgttccctttagtgagggttaattgcgcgcttggcgtaatcatggtcatagctgtttcctgtgtgaaattgttatccgctcacaattccacacaacatacgagccggaagcataaagtgtaaagcctggggtgcctaatgagtgagctaactcacattaattgcgttgcgctcactgcccgctttccagtcgggaaacctgtcgtgccagctgcattaatgaatcggccaacgcgcggggagaggcggtttgcgtattgggcgctcttccgcttcctcgctcactgactcgctgcgctcggtcgttcggctgcggcgagcggtatcagctcactcaaaggcggtaatacggttatccacagaatcaggggataacgcaggaaagaacatgtgagcaaaaggccagcaaaaggccaggaaccgtaaaaaggccgcgttgctggcgtttttccataggctccgcccccctgacgagcatcacaaaaatcgacgctcaagtcagaggtggcgaaacccgacaggactataaagataccaggcgtttccccctggaagctccctcgtgcgctctcctgttccgaccctgccgcttaccggatacctgtccgcctttctcccttcgggaagcgtggcgctttctcatagctcacgctgtaggtatctcagttcggtgtaggtcgttcgctccaagctgggctgtgtgcacgaaccccccgttcagcccgaccgctgcgccttatccggtaactatcgtcttgagtccaacccggtaagacacgacttatcgccactggcagcagccactggtaacaggattagcagagcgaggtatgtaggcggtgctacagagttcttgaagtggtggcctaactacggctacactagaaggacagtatttggtatctgcgctctgctgaagccagttaccttcggaaaaagagttggtagctcttgatccggcaaacaaaccaccgctggtagcggtggtttttttgtttgcaagcagcagattacgcgcagaaaaaaaggatctcaagaagatcctttgatcttttctacggggtctgacgctcagtggaacgaaaactcacgttaagggattttggtcatgagattatcaaaaaggatcttcacctagatccttttaaattaaaaatgaagttttaaatcaatctaaagtatatatgagtaaacttggtctgacagttaccaatgcttaatcagtgaggcacctatctcagcgatctgtctatttcgttcatccatagttgcctgactccccgtcgtgtagataactacgatacgggagggcttaccatctggccccagtgctgcaatgataccgcgagacccacgctcaccggctccagatttatcagcaataaaccagccagccggaagggccgagcgcagaagtggtcctgcaactttatccgcctccatccagtctattaattgttgccgggaagctagagtaagtagttcgccagttaatagtttgcgcaacgttgttgccattgctacaggcatcgtggtgtcacgctcgtcgtttggtatggcttcattcagctccggttcccaacgatcaaggcgagttacatgatcccccatgttgtgcaaaaaagcggttagctccttcggtcctccgatcgttgtcagaagtaagttggccgcagtgttatcactcatggttatggcagcactgcataattctcttactgtcatgccatccgtaagatgcttttctgtgactggtgagtactcaaccaagtcattctgagaatagtgtatgcggcgaccgagttgctcttgcccggcgtcaatacgggataataccgcgccacatagcagaactttaaaagtgctcatcattggaaaacgttcttcggggcgaaaactctcaaggatcttaccgctgttgagatccagttcgatgtaacccactcgtgcacccaactgatcttcagcatcttttactttcaccagcgtttctgggtgagcaaaaacaggaaggcaaaatgccgcaaaaaagggaataagggcgacacggaaatgttgaatactcatactcttcctttttcaatattattgaagcatttatcagggttattgtctcatgagcggatacatatttgaatgtatttagaaaaataaacaaataggggttccgcgcacatttccccgaaaagtgccac

pubb-QFDBD-2x AD*-VP16*-EcR, α-cry:EGFP

ctaaattgtaagcgttaatattttgttaaaattcgcgttaaatttttgttaaatcagctcattttttaaccaataggccgaaatcggcaaaatcccttataaatcaaaagaatagaccgagatagggttgagtgttgttccagtttggaacaagagtccactattaaagaacgtggactccaacgtcaaagggcgaaaaaccgtctatcagggcgatggcccactacgtgaaccatcaccctaatcaagttttttggggtcgaggtgccgtaaagcactaaatcggaaccctaaagggagcccccgatttagagcttgacggggaaagccggcgaacgtggcgagaaaggaagggaagaaagcgaaaggagcgggcgctagggcgctggcaagtgtagcggtcacgctgcgcgtaaccaccacacccgccgcgcttaatgcgccgctacagggcgcgtcccattcgccattcaggctgcgcaactgttgggaagggcgatcggtgcgggcctcttcgctattacgccagctggcgaaagggggatgtgctgcaaggcgattaagttgggtaacgccagggttttcccagtcacgacgttgtaaaacgacggccagtgagcgcgcgtaatacgactcactatagggcgaattgGGTACCCAGAGGTGTAAAAAGTACTCAAAAATTTTACTCAAGTGAAAGTACAAGTACTTAGGGAAAATTTTACTCAATTAAAAGTAAAAGTATCTGGCTAGAATCTTACTTGAGTAAAAGTAAAAAAGTACTCCATTAAAATTGTACTTGAGTATTCTCGAGgtcgacggtatcgataagcttgatatcGAATTACCAGCAAAGTTCTAGAATTTGTCGAAACATTTATGTTATATATTTCCTGAAAAAAATTCTGAGTAAGTTCTTAAGTGTTATTGCCAGCAACATAAACAACAGACGGCAAAATGAATAAATGATAACAAAGCAGTAGGCTTAAATAAACCTAATTTTTATAGGCTGTTCTCTACAACCCTCAAACAGTGATTAGTTTTGTACTTATAAACTTGCCCTTTCATTCATATTTCAAGAAAATTGGTTCAGAAGATCTGGATATTCTAGCAGTTGTTCAAGCTCATGGAGGGATCAGTGACCTGATTCCACAATGACTAGGCCTAATCCAGAAATTAGATGACTGTCAACATAAAAAGGCACAGCACTCACTAGCTGCCCTATATATTTTATTATATTTTACATATATTATTTTATTTATTTAGCTCTGAGTGCTGTACTTTCTGGTTAAAGAAAACTGCTTACAACAGCTAACCTGTACTACCTCAGGCTCAGGGAATTTGGAACAGGTTTGTCTGGTTTGTTTCTTTAACCATGCATGCTTGTTTTCAACTATGGCAACACAGTCACATGGGACATTACAGAAATGATTTGTCGATGACATGCGACTTTTCTTTAATAAAGCGCAAAGATCCCAAAAAGCAAACTTTTAACAAAAATCATATAATTATATTTTCAATCCAGCTTTGTAGCAACTTTGTGCTGCTGTTCACTCAGCAACAGATAGTCAGTATAAGGTCAGTGTGTCTCAAAGCAGTGCCATCTGTTTCACACATTGCGTTCTATATATAAGTGtgctggttgacacgacactgtataaggcctaggctaaaacacaaacaatgtagaatgacactgtgttttttttgtaaacaaatgttgtttttggttaaacatctttgtgaaaacatcctcctgtcatgtatttgctatattcaaatgttaaacccgtgcagaatagaacatatacaaaaaaaaacaacacaacacatttttaaacattattaaatatcaagtattgctggcagttctgtttctgttttacagtaccctttgccacagttctccgcttttcctggtccagattccacaagtctgattcaccaatagcaaagcgaataaacaaccaaagcagccaatcactgcttgtagactgtcctgcgagaccggcccattccagcacattctggaaacttcctttatatgataattataaatacatttaaattattgatacaaaacatgtaattcctagaacataaccatagcaatcattagttttcagggtaattatgtatttttaggatttgactgcggaaagatctggtcatgtgacgtctcatgaacgtcacggccctgggtttctataaatacagtaggactctcgaccatcggcagatttttcgaagaagaagatcagtttcaggagccgtactgttccgtttcaacgcaaatattaacggtaagagcgaatttcctagtttgttttcatgccattctttaaaaccatagcgtattactttaattatagtaaactttcgctttctttattacaagagacgttttgtgttgattctccgcggacattttcggtcagacaatcagaaaatgaccgcggaggaccagtaacttgcattacacgtaagttaaatcttcgtgtattaaaatggttaggttgttaacgtcaaataggttaccgtgtttgcgtgtgatcaggttggttttgttagatttttgtcagtatttttaatttatttgttttagtttatttattttttttgctgaatcatagtttgtgaacaaagaacccggatgttacatacagtacagccgccatgttacagagagttataacttaatcattttaaaaataattttgccttacttttagtttgtcatgttgagaaatgaggaaatgttaaaatgaggaaatatccaattaatttaatatatcaaaataatccatgattacaatgcactgaactggagaaaattaagatgttttctagtgtcatgaaacaaatgtaagagatgtacattgtagatgttttatgtcaagaattggctagttgatgcagcatactggcgatactcagttgtaataacagtaacgttacatgttaatagactactgagtatgctgttctgtctatgtatgctctgtaagctacgagaaggacttttttaaacagtaaagggtgcaatatttttacaaattgaattaaataaaggctgtctattaagtaatatgcttgatatttttcttacttgatcggaaataagaaaaaatataaacgttgttgctctaaaaatcctagttcagtttagccaaccacaaatacctttttgttcctccaacagtttttttttcttctctataatatttggcagtctatagtactccaaatgtttccccacagtctaactaattggtacagccaaaatcatgacacttattgcaataataattttggttcattggcattgttgatagcctgtgccactaatatggtcgattgatcatgcttcaggaagaaaactatattgtttgatgtaagattattaaatcttcacctgcttccattacaaactattcccatcttattGAATTCTGGTATGTCTTAAAGGATTAGTTCACTTCCCAAATCAAAATTTACTTAGCCTTTTTTCATCCATGATCCCTTTTTTTCATCATTAATGAAGAAATTGTTTTTGAAAAAGTTTCAAGATTTTTTTCTCTATATTGTGGAGCTTGTTAGTTTAAAATTCCAAAATGCAATATGTGGCTTCAAATGGTTCTAAATGATCCCAGTCAAGGAATAACAGTCTTATCTAATGAAACCATTAGACCTTTAAAAATAAAAAAAATAAAAGTATTTATTTTTAAATGACTGAGTGATTAAGTTGAATTTCAGCGTTTCCTTACTGTGTAGAAGTCCTTCCTTACTGGCCCCACCCTTTGGTTCTCTGCCAATCTGCTACCTAATGTAATGTTGTGGAACATTATTATTCTTTATTTCTTAATTTTTTATTTTTTATTTTAAAACAATGTAAACTGCACAGATGTGCAGTTTGTTTAAAATGGCCAATGCTTTGGAAATGCATGACATAATTAGATTTCATGATGCACAAAGCCAAATCTCAGAGCTTGTGCAAAATGAGCTATCATTTCACTAGGTAAGACCCTAAATTTTCATATAGGATCATTTGGACAATTTTGCTGCAGGTAAAATGCATTCTATAGTCCACTGTCAGCCATTGTTTTGGATAGTATTTATTTTTCTCTACAAGTATAGTCAATAGTTTTCTATTATTTTAAAGGTTTGTAACATTTAAGGGTGACCAAATGCAAAGTAAAATTTCATTTTCGGGTGAACTATCTCGTTTAACATGGGAGAAGTGCAAAACATACATTATTGGCTAGAACATTGTAGTATTTTTTAAATGGAAATGTGTGATTGCTAATCTTACTTTGAATTTGTTTACAGgaattcctgcagcccggggCCAACATGCCGCCTAAACGCAAGACACTCAATGCCGCTGCCGAAGCCAATGCCCACGCTGATGGCCATGCTGATGGCAATGCTGATGGTCATGTCGCTAACACTGCAGCAAGCAGCAACAACGCCCGTTTTGCGGACTTGACCAACATTGACACACCCGGCCTCGGCCCTACCACGACGACGTTACTTGTCGAGCCCGCTCGTTCGAAACGCCAGAGAGTCTCGAGGGCCTGTGATCAGTGTCGAGCTGCACGTGAAAAGTGTGATGGAATCCAGCCGGCTTGCTTCCCCTGTGTGTCGCAGGGCCGGTCGTGTACCTACCAGGCCAGTCCCAAGAAGCGAGGAGTCCAGACGGGCTACATCCGCACTCTCGAACTGGCTCTGGCTTGGATGTTCGAGAACGTTGCCCGCAGCGAGGACGCCCTCCACAATCTTTTGGTCCGTGATGCTGGCCAGGGCAGCGCTCTCCTGGTCGGCAAAGACTCGCCTGCTGCAGAACGCCTGCATGCAAGATGGGCGACGAGTCGAGTCAACAAAAGCATCACCCGTCTTCTCTCAGGTCAGGCCGCACAAGATCCATCTGAAGACGGCCAATCCCCGTCCGAAGACATAAATGTCCAAGATCAGGCCGCACAAGATCCATCTGAAGACGGCCAATCCCCGTCCGAAGACATAAATGTCCAAGATGAGTTCCTGGATATGGCCGACTTCGAGTTTGAGCAGATGTTTACCGATGCCCTTGGAATTGACGAGTACGGTGGGCTGCAGATGAGGCCTGAATGTGTCATACAGGAGCCCAGTAAAAATAAAGACAGGCAAAGACAAAAGAAAGACAAAGGAATATTATTACCTGTTAGTACGACCACAGTCGAAGACCACATGCCCCCGATCATGCAATGTGATCCACCTCCGCCCGAGGCCGCCAGGATTCACGAAGTCGTCCCGAGGTATCTTTCGGAGAAGCTGATGGAGCAGAACAGGCAGAAGAACATACCACCATTGTCGGCGAATCAGAAGTCTCTGATCGCGAGGCTCGTGTGGTACCAGGAGGGATATGAGCAGCCCTCCGACGAGGATCTCAAAAGAGTAACGCAGACTTGGCAGTCGGATGAAGAGGACGAGGAATCCGATCTACCCTTCCGCCAGATCACGGAGATGACGATCTTAACGGTCCAGTTGATCGTCGAGTTCGCCAAGGGTCTACCGGGCTTTTCGAAGATATCACAGTCTGATCAAATCACCTTATTAAAAGCCTCGTCCAGCGAGGTGATGATGCTGCGGGTGGCGAGGCGATACGACGCCGCGTCCGACAGCATTCTGTTCGCCAACAACAAGGCGTACACGCGCGACAACTACCGCAAGGCGGGCATGGCCGAGGTCATCGAAGACCTCCTACACTTCTGCCGGTGCATGTTCGCGATGGGCATGGACAATGTGCACTTTGCACTGCTCACGGCCATCGTTATATTCTCAGATCGGCCCGGGCTCGAGCAGCCGTCGCTGGTAGAAGAGATCCAGAGATACTACCTGAACACGTTGCGAATTTACATCATCAACCAGAACAGCGCGTCGTCGCGCTGCGCCGTGATCTACGGCAGGATCCTGAGCGTGCTGACCGAGCTACGCACGCTCGGCACGCAAAACTCCAACATGTGCATCTCGCTGAAGCTGAAGAACAGGAAGCTGCCGCCGTTCCTCGAGGAGATCTGGGACGTGGCGGAGGTGGCCACGACGCATCCCACGGTGCTGCCGCCCACCAACCCGGTGGTGCTATAGgggggatccactagtaattaagtctcagccaccgttaactgaacatgtcaaaacctgtggagactgttgagatttgatgttctgaaaagataaagcctataaataaaatgttgcccaaatttcctgcctgatgtttttctttgtctttgctacatggctttgctgctcggatcggctcactctgtgtatgccacgttcactttgtactctccttctcacggtaggtttattatttttagatgtgcagttagtttctgtgaaataacacaccacacactgatattgtctgtgcattgacttggtgagtgcacattgtttttgatcttgacatatttatatttgattgatcaggtgaactgtgtgaatctaaagtgctccatacagatgttctgcattgaaaatattctcattttattagtggaagtgagtgtatgccacatccaatcaatttcagcaaacaccccagtatgatttaatgcaaaaaaatgaaggtatcaaacacgcattactactttgcagttaaatatttaacatttattccaacacgaaaaaaagcagtaaataacactttgacaaacacgtcaggacatcttatttttgtcaccctcacaggcaatttagtataatatattatatatatatatatatcatataataatattcagtataatatatatatatatatcatattataatattcagtataatataaaacacaaacacatatatgtataatataatataacatttttatttattgagatgcctctatggaccgtgttataagaagtaaagatcaggagaagtaaacatgaagtgtaattatgaatactgatgttaaattaagctatgatgagttttcactgttaatttaccatctcaattaaatgttgatgcctccatgaccaagttaagcagatgagactgagacaactgtagaagacaagatgttcactttgctgaatatagctggcttgacagttatctatgactctataaatatatatatatttttttttttataaaatgatttatttataactatatatccatttctcagacaggtgcttcatatccctcactcccgtagctgtccatgctggatctgtccccgttgtttttaaaaagctaaataagttattaacatgactgcatccagcgagccaaacctgtctggtgtacagctaccagagaagcttgagatcctagtcaccgGCGGCCGCTAATATGGCaataaacgatcttcagagaaacttgcattggtaaatagtttcagcctatgcatatgtagttattcaaactgtaatgactatcttacagtattatatgtttataatagtttgatccaataaatccagatttatatgcttttcaacataaaattgtgaaatgcacagaatgtacatatacaatacatttcctacaataatgctctcttttctattaatagtgtgcattcagtgcaggatgttatgtatttattttctgtagtgcaaactttttcaaagaattttccagtctaagcttttcagtcaagaaaaaaaaaagcgattgattatctgtgatatttaaaaaaattagctcttaaatctggtgaaccaagtggccacaacacattaaatactgacgttctacagccatgtagcttcatctggtctggtttgttttggcaggcacttattttccggtgatagttggccacaagagtattgtctgagccattcagtgctagactgtcattctcaggtcagagtccaccccgctgattctgctgacaacactgttcccaccatgagataatgccattccagagagatccatttgtaagcccctctttctgcagcacaggtatataaccaggggtctgcctccactaaggccggcacacatcatttggggatctttgtactgtGGATCCACCGGTCGCCACCATGGTGAGCAAGGGCGAGGAGCTGTTCACCGGGGTGGTGCCCATCCTGGTCGAGCTGGACGGCGACGTAAACGGCCACAAGTTCAGCGTGTCCGGCGAGGGCGAGGGCGATGCCACCTACGGCAAGCTGACCCTGAAGTTCATCTGCACCACCGGCAAGCTGCCCGTGCCCTGGCCCACCCTCGTGACCACCCTGACCTACGGCGTGCAGTGCTTCAGCCGCTACCCCGACCACATGAAGCAGCACGACTTCTTCAAGTCCGCCATGCCCGAAGGCTACGTCCAGGAGCGCACCATCTTCTTCAAGGACGACGGCAACTACAAGACCCGCGCCGAGGTGAAGTTCGAGGGCGACACCCTGGTGAACCGCATCGAGCTGAAGGGCATCGACTTCAAGGAGGACGGCAACATCCTGGGGCACAAGCTGGAGTACAACTACAACAGCCACAACGTCTATATCATGGCCGACAAGCAGAAGAACGGCATCAAGGTGAACTTCAAGATCCGCCACAACATCGAGGACGGCAGCGTGCAGCTCGCCGACCACTACCAGCAGAACACCCCCATCGGCGACGGCCCCGTGCTGCTGCCCGACAACCACTACCTGAGCACCCAGTCCGCCCTGAGCAAAGACCCCAACGAGAAGCGCGATCACATGGTCCTGCTGGAGTTCGTGACCGCCGCCGGGATCACTCTCGGCATGGACGAGCTGTACAAGTAAAgcggccggccgcGACTCTAGATCATAATCAGCCATACCACATTTGTAGAGGTTTTACTTGCTTTAAAAAACCTCCCACACCTCCCCCTGAACCTGAAACATAAAATGAATGCAATTGTTGTTGTTAACTTGTTTATTGCAGCTTATAATGGTTACAAATAAAGCAATAGCATCACAAATTTCACAAATAAAGCATTTTTTTCACTGCATTCTAGTTGTGGTTTGTCCAAACTCATCAATGtatctaagccgcggtgGAGCTCAAGTGATCTCCAAAAAATAAGTACTTTTTGACTGTAAATAAAATTGTAAGGAGTAAAAAGTACTTTTTTTTCTAAAAAAATGTAATTAAGTAAAAGTAAAAGTATTGATTTTTAATTGTACTCAAGTAAAGTAAAAATCCCCAAAAATAATACTTAAGTACAGTAATCAAGTAAAATTACTCAAGTACTTTACACCGAGCTCcagcttttgttccctttagtgagggttaattgcgcgcttggcgtaatcatggtcatagctgtttcctgtgtgaaattgttatccgctcacaattccacacaacatacgagccggaagcataaagtgtaaagcctggggtgcctaatgagtgagctaactcacattaattgcgttgcgctcactgcccgctttccagtcgggaaacctgtcgtgccagctgcattaatgaatcggccaacgcgcggggagaggcggtttgcgtattgggcgctcttccgcttcctcgctcactgactcgctgcgctcggtcgttcggctgcggcgagcggtatcagctcactcaaaggcggtaatacggttatccacagaatcaggggataacgcaggaaagaacatgtgagcaaaaggccagcaaaaggccaggaaccgtaaaaaggccgcgttgctggcgtttttccataggctccgcccccctgacgagcatcacaaaaatcgacgctcaagtcagaggtggcgaaacccgacaggactataaagataccaggcgtttccccctggaagctccctcgtgcgctctcctgttccgaccctgccgcttaccggatacctgtccgcctttctcccttcgggaagcgtggcgctttctcatagctcacgctgtaggtatctcagttcggtgtaggtcgttcgctccaagctgggctgtgtgcacgaaccccccgttcagcccgaccgctgcgccttatccggtaactatcgtcttgagtccaacccggtaagacacgacttatcgccactggcagcagccactggtaacaggattagcagagcgaggtatgtaggcggtgctacagagttcttgaagtggtggcctaactacggctacactagaaggacagtatttggtatctgcgctctgctgaagccagttaccttcggaaaaagagttggtagctcttgatccggcaaacaaaccaccgctggtagcggtggtttttttgtttgcaagcagcagattacgcgcagaaaaaaaggatctcaagaagatcctttgatcttttctacggggtctgacgctcagtggaacgaaaactcacgttaagggattttggtcatgagattatcaaaaaggatcttcacctagatccttttaaattaaaaatgaagttttaaatcaatctaaagtatatatgagtaaacttggtctgacagttaccaatgcttaatcagtgaggcacctatctcagcgatctgtctatttcgttcatccatagttgcctgactccccgtcgtgtagataactacgatacgggagggcttaccatctggccccagtgctgcaatgataccgcgagacccacgctcaccggctccagatttatcagcaataaaccagccagccggaagggccgagcgcagaagtggtcctgcaactttatccgcctccatccagtctattaattgttgccgggaagctagagtaagtagttcgccagttaatagtttgcgcaacgttgttgccattgctacaggcatcgtggtgtcacgctcgtcgtttggtatggcttcattcagctccggttcccaacgatcaaggcgagttacatgatcccccatgttgtgcaaaaaagcggttagctccttcggtcctccgatcgttgtcagaagtaagttggccgcagtgttatcactcatggttatggcagcactgcataattctcttactgtcatgccatccgtaagatgcttttctgtgactggtgagtactcaaccaagtcattctgagaatagtgtatgcggcgaccgagttgctcttgcccggcgtcaatacgggataataccgcgccacatagcagaactttaaaagtgctcatcattggaaaacgttcttcggggcgaaaactctcaaggatcttaccgctgttgagatccagttcgatgtaacccactcgtgcacccaactgatcttcagcatcttttactttcaccagcgtttctgggtgagcaaaaacaggaaggcaaaatgccgcaaaaaagggaataagggcgacacggaaatgttgaatactcatactcttcctttttcaatattattgaagcatttatcagggttattgtctcatgagcggatacatatttgaatgtatttagaaaaataaacaaataggggttccgcgcacatttccccgaaaagtgccac
