# Supplementary material for: IQ-Switch is a QF-based innocuous, silencing-free, and inducible gene switch system in zebrafish
Source: Commun Biol. 2021 Dec 16;4:1405. doi: 10.1038/s42003-021-02923-3 (PMC8677817; doi:10.1038/s42003-021-02923-3)
Supplement: Supplementary file 5 — Reporting Summary [file 42003_2021_2923_MOESM5_ESM.pdf]

nature research

Corresponding author(s): DBPR, COMMSBIO-21-1364-T

Last updated by author(s): Jun 2, 2021

Reporting Summary

Nature Research wishes to improve the reproducibility of the work that we publish. This form provides structure for consistency and transparency in reporting. For further information on Nature Research policies, see our Editorial Policies and the Editorial Policy Checklist.

Statistics

For all statistical analyses, confirm that the following items are present in the figure legend, table legend, main text, or Methods section.

n/a	Confirmed	
☐	☒	The exact sample size (*n*) for each experimental group/condition, given as a discrete number and unit of measurement
☐	☒	A statement on whether measurements were taken from distinct samples or whether the same sample was measured repeatedly
☐	☒	The statistical test(s) used AND whether they are one- or two-sided<br>*Only common tests should be described solely by name; describe more complex techniques in the Methods section.*
☐	☒	A description of all covariates tested
☐	☒	A description of any assumptions or corrections, such as tests of normality and adjustment for multiple comparisons
☐	☒	A full description of the statistical parameters including central tendency (e.g. means) or other basic estimates (e.g. regression coefficient) AND variation (e.g. standard deviation) or associated estimates of uncertainty (e.g. confidence intervals)
☐	☒	For null hypothesis testing, the test statistic (e.g. *F*, *t*, *r*) with confidence intervals, effect sizes, degrees of freedom and *P* value noted<br>*Give P values as exact values whenever suitable.*
☐	☒	For Bayesian analysis, information on the choice of priors and Markov chain Monte Carlo settings
☐	☒	For hierarchical and complex designs, identification of the appropriate level for tests and full reporting of outcomes
☐	☒	Estimates of effect sizes (e.g. Cohen's *d*, Pearson's *r*), indicating how they were calculated

Our web collection on statistics for biologists contains articles on many of the points above.

Software and code

Policy information about availability of computer code

Data collection	no software was used
Data analysis	Microsoft excel, Bio-Rad CFX Manager™ 3.1 software

For manuscripts utilizing custom algorithms or software that are central to the research but not yet described in published literature, software must be made available to editors and reviewers. We strongly encourage code deposition in a community repository (e.g. GitHub). See the Nature Research guidelines for submitting code & software for further information.

Data

Policy information about availability of data

All manuscripts must include a data availability statement. This statement should provide the following information, where applicable:

- Accession codes, unique identifiers, or web links for publicly available datasets
- A list of figures that have associated raw data
- A description of any restrictions on data availability

The authors declare that [the/all other] data supporting the findings of this study are available within the paper [and its supplementary information files].

Field-specific reporting

Life sciences study design

All studies must disclose on these points even when the disclosure is negative.

Sample size	All experiments were carried out at least in triplicates. The exact number of zebrafish embryos used in this study was indicated in the main text and Figures.
Data exclusions	No data was excluded from alalysis
Replication	All experiments were carried out at least in triplicates and all attempt to replication was successful.
Randomization	All the experiments were carried out with proper control groups with an unbiased manner. All the zebrafish embryos used in this study was collected randomly without any bias.
Blinding	During data collection and analysis, we all were allocated in blinded condition for grouping samples.

Reporting for specific materials, systems and methods

We require information from authors about some types of materials, experimental systems and methods used in many studies. Here, indicate whether each material, system or method listed is relevant to your study. If you are not sure if a list item applies to your research, read the appropriate section before selecting a response.

Materials & experimental systems

n/a	Involved in the study	
☐	☒	Antibodies
☐	☒	Eukaryotic cell lines
☒	☐	Palaeontology and archaeology
☐	☒	Animals and other organisms
☒	☐	Human research participants
☒	☐	Clinical data
☒	☐	Dual use research of concern

Methods

n/a	Involved in the study	
☒	☐	ChIP-seq
☒	☐	Flow cytometry
☒	☐	MRI-based neuroimaging

Antibodies

Antibodies used	anti-Flag (M2, Sigma-Aldrich, F3165), anti-Myc (Santa Cruz Biotechnology, sc-40), anti-Myc (Millipore, 06-549)
Validation	anti-Flag (M2, Sigma-Aldrich, F3165) : https://www.sigmaaldrich.com/catalog/product/sigma/f3165?lang=ko&region=KR&gclid=EAIaIQobChMInuTo-rv48AIVUgVgCh1wzQ6REAAYAiAAEgJ7TfD_BwE<br>anti-Myc (Santa Cruz Biotechnology, sc-40) : https://www.scbt.com/p/c-myc-antibody-9e10<br>anti-Myc (Millipore, 06-549) : https://www.merckmillipore.com/KR/ko/product/Anti-Myc-Tag-Antibody,MM_NF-06-549

Eukaryotic cell lines

Policy information about cell lines

Cell line source(s)	HEK293 and Cos7 from ATCC (https://www.atcc.org/en/cell-products#t=productTab&numberOfResults=12)
Authentication	All cell lines used here are commercially available in elsewhere. We obtained the cell lines from ATCC.
Mycoplasma contamination	All cell lines tested were not contaminated by mycoplasma
Commonly misidentified lines (See ICLAC register)	None

Animals and other organisms

Policy information about studies involving animals; ARRIVE guidelines recommended for reporting animal research

Laboratory animals	Zebrafish, AB line
Wild animals	None
Field-collected samples	None

Ethics oversight	Zebrafish care were performed in accordance with guidelines from the Korea Research Institute of Bioscience and Biotechnology (KRIBB) and Chungnam National University (201903-CNU-007).

Note that full information on the approval of the study protocol must also be provided in the manuscript.
